# Stripping away ion hydration shells in electrical double-layer formation: Water networks matter

**DOI:** 10.1073/pnas.2108568118

**Published:** 2021-11-15

**Authors:** Serena R. Alfarano, Simone Pezzotti, Christopher J. Stein, Zhou Lin, Federico Sebastiani, Sarah Funke, Claudius Hoberg, Inga Kolling, Chun Yu Ma, Katja Mauelshagen, Thorsten Ockelmann, Gerhard Schwaab, Li Fu, Jean-Blaise Brubach, Pascale Roy, Martin Head-Gordon, Kristina Tschulik, Marie-Pierre Gaigeot, Martina Havenith

**Affiliations:** ^a^Lehrstuhl für Physikalische Chemie II, Ruhr–Universität Bochum 44780 Bochum, Germany;; ^b^Université Paris-Saclay, Univ Evry, CNRS, LAMBE UMR8586, Evry-Courcouronnes 91025, France;; ^c^Department of Chemistry, University of California, Berkeley, CA 94720;; ^d^Theoretische Physik, Center for Nanointegration Duisburg-Essen, Universität Duisburg-Essen 47048 Duisburg, Germany;; ^e^Joint Center for Artificial Photosynthesis, Lawrence Berkeley National Laboratory, Berkeley, CA 94720;; ^f^Université de Lyon, Université Claude Bernard Lyon 1, CNRS, Institut Lumière Matière F-69622 Villeurbanne, France;; ^g^Synchrotron Soleil, AILES beamline, L’Orme des Merisiers 91192 Gif sur Yvette Cedex, France;; ^h^Faculty of Chemistry and Biochemistry, Chair of Analytical Chemistry II, Ruhr University Bochum 44780 Bochum, Germany

**Keywords:** electrochemistry, double layer, *operando*, hydrogen bonding, electrolyte

## Abstract

The double layer at the solid/electrolyte interface is a key concept in electrochemistry. Here, we present an experimental study combined with simulations, which provides a molecular picture of the double-layer formation under applied voltage. By THz spectroscopy we are able to follow the stripping away of the cation/anion hydration shells for an NaCl electrolyte at the Au surface when decreasing/increasing the bias potential. While Na^+^ is attracted toward the electrode at the smallest applied negative potentials, stripping of the Cl^−^ hydration shell is observed only at higher potential values. These phenomena are directly measured by THz spectroscopy with ultrabright synchrotron light as a source and rationalized by accompanying molecular dynamics simulations and electronic-structure calculations.

## Significance

For centuries the double layer at the solid/electrolyte interface has been a central concept in electrochemistry. Today, it is still crucial for virtually all renewable energy storage and conversion technologies. Here, the double-layer formation is probed by THz spectroscopy with ultrabright synchrotron light as a source. Our results capture the molecular details of double-layer formation at positively/negatively charged Au electrodes for an NaCl electrolyte. We reveal a contrasting response applying positive versus negative bias, which is dictated by the interfacial water network and rationalized by accompanying molecular dynamics simulations and electronic-structure calculations. While Na^+^ is directly attracted toward the negatively charged electrode, stripping of the Cl^−^ hydration shell is observed only at larger potential values.

One of the most challenging global frontiers is the economic transition toward renewable energy technologies and recycling of waste into valuable chemicals. Most routes to tackle these challenges and develop “green” processes involve electrochemistry. These range from battery, supercapacitor, and fuel cell technologies (1), solar cells (2), and electrochemical water splitting (3) all the way to using electric power for selective reduction of CO_2_ to form synthetic renewable fuels and valuable chemicals (4). Each of these applications involves electron transfer across the electrode/solution interface and is therefore governed by interfacial chemistry. Thus, all major efforts rely on increasing the speed and selectivity of interfacial reactions. This holds for electrocatalysis as much as for batteries.

The fundamental principles governing interfacial chemistry were established decades ago. Marcus theory and the Butler–Volmer formalism describe the general principles of electrochemical reaction kinetics, based either on considering the solvation of reactants, intermediates, and products or the activated complex formed upon electron transfer, respectively. In parallel, the Mott–Schottky equation for the depletion layer facilitates the prediction of the capacitive behavior at the semiconductor/electrolyte interface on a macroscopic basis (5). In reality, however, very little is known at the molecular level about the structure and solvation state of the reacting species and the way they are activated directly at the electrode, due to the lack of a microscopic description of the double layer arising at the solid/electrolyte interface. Several experiments (6–8) showed the validity of the Gouy–Chapman–Stern–Grahame (GCS) model for the description of solid/electrolyte interfaces at a macroscopic level. According to the GCS model (9), the charged electrode is in contact with the inner Helmholtz plane (IHP), composed of dehydrated immobile ions specifically bound to the surface. The outer Helmholtz plane (OHP), consisting of hydrated ions, adjoins the IHP, which is in turn followed by a diffusive layer with mobile ions. *Per contra*, on the microscopic scale, the interfacial solvent structure and ion solvation in the IHP/OHP are not explicitly treated in the GCS model, and deviations from the model have been reported (10, 11). Whatever the electrocatalytic process, the reactants must approach the surface to react, i.e., they have to penetrate the electric double layer (EDL). A microscopic understanding of the EDL and the role of the solvent (water in most electrochemical applications as of today) in mediating ion–surface interactions is therefore of fundamental importance for future applications.

This requires new experimental techniques that are able to selectively probe the EDL and simultaneously provide molecular-level information, under *operando* (i.e., operating or reaction) electrochemical conditions. In the past two decades, a number of theoretical (12–17) and experimental (18–27) studies have been performed to explore the microscopic structure of the electrochemical double layer. It is still an experimental challenge to probe interfaces under *operando* conditions, due to difficulties in regulating all the variables involved in electrochemical reactions (28, 29), such as controlling surface structure and mass transport. Charge transfer at an unperturbed double layer has been characterized by an electrochemical probe (30). Using infrared (IR) spectroscopy, Yamakata et al. (24) investigated modifications in the ion hydration shells on a CO-covered Pt electrode. Local and intramolecular mode probes have been used in sum frequency generation experiments at aqueous interfaces (31) or in surface enhanced Raman scattering/Stark effect spectroscopies (32).

As discussed in more detail in ref. 15, water molecules within the adlayer preferentially lie flat on the Au surface, forming a two-dimensional hydrogen bond (2D-HB) network composed of HBs oriented parallel to the surface. As a consequence, only a few HBs form between the adlayer and the second water layer, creating a soft liquid–liquid interface (33). When an increasing negative potential is applied, interfacial water molecules gradually reorient their H atoms toward the gold surface, hence disrupting the 2D-HB network in the adlayer (15, 21). These structural changes affect the EDL formation, since the adsorption of ions at the metal surface was shown to require a free energy cost due to perturbation of the adlayer structure and removal of one (or more) water molecule(s) from it (13).

In this work, we focus on changes in the water network, such as the formation and stripping away of the ions’ hydration shell during the formation of the EDL. Any changes in the HB network as well as ion hydration can be sensitively probed by THz spectroscopy, i.e., in the intermolecular fingerprint region between 10 and 700 cm^−1^ (34). The low-frequency absorption spectrum of bulk water is well known (34–37), as are the low-frequency spectra of bulk aqueous salt solutions (38, 39). In particular, experimental THz fingerprints, i.e., characteristic absorption features whose intensities scale linearly with electrolyte concentration, have been identified for anions and cations. These features have been assigned to so-called rattling modes for the case of strongly hydrated ions within their hydration cages, or in the case of weakly hydrated ions as vibrationally induced charge fluctuations (38).

We present a fingerprint of the double-layer formation of an NaCl electrolyte solution at a gold surface under applied bias potential using the ultrabright synchrotron Soleil as a low-frequency radiation source. Any modification of the ions’ hydration environment upon application of applied voltage will be identified by the change from their well-known bulk THz fingerprints. Our objective is to use new THz experiments and accompanying simulations to uncover similarities and differences in the response of hydrated Na^+^ and Cl^−^ ions to the applied bias associated with EDL formation at the gold electrode.

## Results

We used a 10 mM NaCl solution and applied a positive/negative potential at the gold (Au)–liquid interface. Following the pioneering work of Nemes et al. (40), we adopted the electrochemical cell developed at AILES (Soleil Facility) (41), consisting of an Au grid as working electrode and a thin platinum foil as counterelectrode. Since no Faradaic reactions were driven (see *SI Appendix*, Fig. S2), we did not employ a reference electrode to prevent concentration alterations of the sample. The potential values are reported relative to a reference potential (V_ref_), at which zero current is measured and no external bias is applied (open circuit potential; see *SI Appendix*, section S8 for details and conversion to an Ag/AgCl reference electrode).

**Fig. 1.**
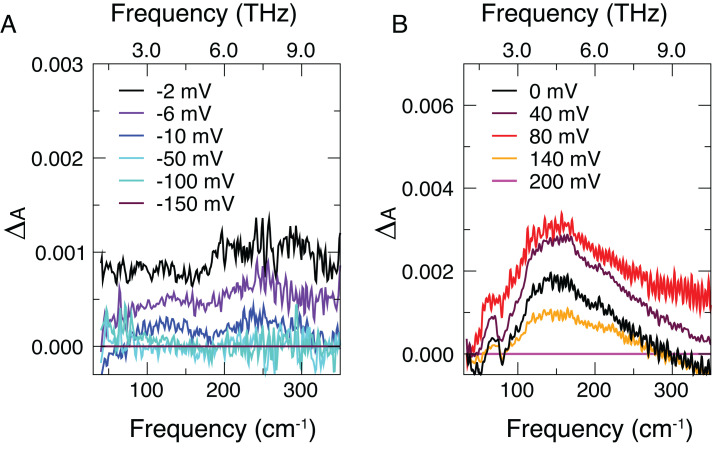
Absorption spectra as a function of the applied potential. (*A*) Spectra acquired during negative potential series for six selected bias voltages. Each spectrum, ΔA(ν; V_i_), is referenced to the spectrum acquired at −150 mV. (*B*) Spectra acquired during positive potential series for five selected voltages. Each spectrum, ΔA(ν; V_i_), is referenced to the spectrum acquired at +200 mV. The total set of spectra upon bias potential application is displayed in *SI Appendix*, Figs. S7 and S8. Following the curves from small voltage to large voltage shows changes in spectra as the EDL is formed by applied bias; note the nonmonotonic behavior for positive bias.

We recorded absorption THz spectra in the 50- to 350-cm^−1^ region, varying the voltage in 20-mV steps from 0 V to +200 mV for the positive voltages, and at the following values for the negative voltages: −2, −4, −6, −8, −10, −20, −30, −50, −100, and −150 mV. We confirmed that background variations are negligible by taking a spectrum at the reference potential V_ref_ immediately after acquisition of each spectrum, A(ν; V_i_), at the potential V_i_. Fig. 1 shows the difference spectra, ΔA(ν; V_i_) = A(ν; V_i_) − A(ν; V_max_), obtained by referencing each spectrum (corrected against its background reference) at potential V_i_ to the spectrum at the lowest or highest applied bias (V_max_) for the negative (Fig. 1*A*) or positive (Fig. 1*B*) potential series, respectively. Henceforth we refer to ΔA(ν; V_i_) as the spectrum. This choice allows clear visualization of the trends with applied voltage. A crucial advantage is that, in the difference spectra, any spectral contribution that does not change with applied voltage does not contribute by construction. Therefore, the intensity of the difference spectra only arises from the region where the effect of applied voltage is nonzero, i.e., in the EDL. The signal-to-noise ratio depends on the probed interfacial volume and it is estimated to be 2·10^−4^ (cf. *SI Appendix*, section S3). All details about the experimental setup are given in *SI Appendix*, sections S1 through S9.

For negative bias (i.e., EDL enriched with Na^+^ ions), the spectral intensity sharply decreases for low potentials (from −2 mV to −10 mV) and saturates around zero already at −50 mV. The spectral intensity does not change from −50 mV up to the most negative potential. A strikingly different trend emerges for positive voltages (i.e., EDL enriched with Cl^−^ ions): The spectral intensity initially increases with increasing potential from 0 mV to +80 mV and starts to decrease only at higher potential values. The monotonic changes in spectral data for Na^+^ in the EDL versus nonmonotonic behavior for Cl^−^ suggest intriguingly different responses of the hydrated ions to applied bias.

In order to decipher the reason behind the different behavior with negative and positive potentials and to unveil the spectral components responsible for the observed trends, we performed a principal component analysis (PCA). As an overview, for both positive and negative potential series a matrix ***M*** is constructed, each row containing one of the measured difference spectra, ΔA(ν; V_i_), and subsequently diagonalized in order to obtain an orthonormal basis set carrying the fundamental effect of the applied potential on the spectral features. In practice, each spectrum is represented as a linear combination of potential-independent partial spectral components (principal components, PCs) weighted by the potential-dependent scores (eigenvalues). While a standard fitting procedure requires assumptions on the number of the bands in a given spectral region, their position, and shape, the advantage of PCA is that it does not depend on any a priori knowledge. However, the extracted spectral components still need to be associated with meaningful physical observables. The scores obtained from the PCA are reported in *SI Appendix*, Fig. S1.

**Fig. 2.**
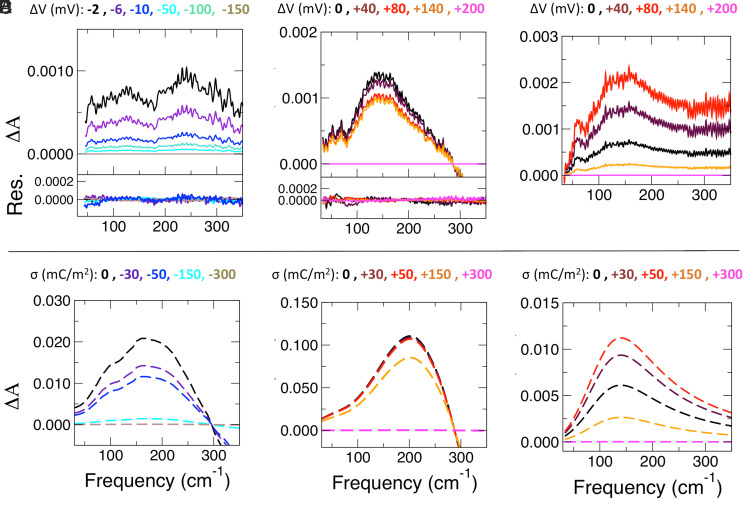
Experimental and theoretical partial spectral components as a function of negative and positive applied potential and surface charge. (*A*, *Top*) Experimental partial spectra reconstructed for PCA for discrete negative applied potentials, attributed to Na^+^ hydration. (*A*, *Bottom*) Residuals derived from the subtraction of the partial spectra from the total spectra in Fig. 1*A*. (*B*) Experimental partial spectra reconstructed for PCA for discrete positive applied potentials, attributed to Cl^−^ hydration. (*Bottom*) Residuals. (*C*) Experimental partial spectra reconstructed for PCA for discrete positive applied potentials, attributed to the interfacial water network. (*D*) Simulated spectrum of hydrated Na^+^ from ref. 39 multiplied by the variation in the number of hydration water around Na^+^ cations as a function of the negative surface charge as deduced in the present MD simulations. (*E*) Simulated spectrum of hydrated Cl^−^ ion from ref. 39 multiplied by the variation in the number of hydration waters around Cl^−^ as a function of the positive surface charge as deduced in the present MD simulations. (*F*) HB stretch mode of water molecules hydrating a hydrophobic (alcohol) surface, as in ref. 43, multiplied by the number of HBs of the 2D-HB network as a function of the positive surface charge as deduced in the present MD simulations. We point out that a feature at 70 cm*^−^*^1^ is observed in all experimental spectra (*A*, *B*, and *C*), which is related to the spectrum of the synchrotron light, used as probing source. Increasing surface charge is associated with increasing voltage, though not necessarily via a 1:1 mapping.

As shown in Fig. 2*A*, a single spectral component is sufficient to describe the spectral changes with negative potentials, while two independent components are found for ΔA(ν; V_i_) positive potentials (Fig. 2 *B* and *C*). The residuals, obtained by subtracting the sum of the corresponding spectral components from the total spectrum (in Fig. 1) at each voltage (Fig. 2*A* for negative potentials and Fig. 2 *B* and *C* for positive potentials), demonstrate that this low number of independent components is indeed sufficient to reproduce the full spectrum. Based on comparison to the well-known experimental THz fingerprints of the bulk electrolytes (38, 39), we assign the absorption feature between 80 and 200 cm^−1^ of the PC of the negative potential series (displayed in Fig. 2*A*) to the rattling modes of Na^+^ (which in bulk electrolyte are centered at 80 cm^−1^ and 150 cm^−1^). In a similar way, the feature at ∼150 cm^−1^ of one of the two significant PCs derived from the positive potential series (in Fig. 2*B*) is assigned to the rattling mode of Cl^−^ (centered at 190 cm^−1^). As this feature reflects differences relative to bulk Cl^−^, the experiments imply that hydrated Cl^−^ near the surface exhibits a red-shifted THz fingerprint at 0 V. However, we are left with an additional feature at ∼250 cm^−1^ in Fig. 2*A*, as well as an additional PC (displayed in Fig. 2*C*) for the positive potential series. We propose that these contributions are due to changes in the interfacial water network, as will be discussed further on.

The experimental spectra are compared to theoretical results obtained from classical molecular dynamics (MD) simulations of positively and negatively charged Au–electrolyte interfaces (17 simulations with surface charge, σ, values from −300 mC/m^2^ to +300 mC/m^2^; see *Methods* for details). In the following, the potential dependence of the three PCs is analyzed and systematically compared with simulated spectral features. The theoretical spectra are displayed in Fig. 2 *D–F*, as a function of increasing/decreasing surface charge. For each of the three experimentally derived PCs discussed above (Fig. 2 *A*–*C*), we assign a theoretical spectral component (Fig. 2 *D–F* ). This allows us to map the spectral changes to the atomistic processes at the EDL under applied bias.

First, the experimental spectral component for negative potentials in Fig. 2*A* is compared and assigned to the theoretical THz spectrum of solvated Na^+^ as a function of the Au surface charge (σ) in Fig. 2*D*. As detailed in *Methods*, the spectra in Fig. 2*D* are obtained by multiplying the theoretical spectrum of bulk solvated Na^+^ (39) by a σ-dependent weighting factor, chosen to be the change in the number of hydration water molecules in the first solvation shell of Na^+^ in our EDL MD simulations versus bulk. The calculated spectrum of bulk solvated Na^+^ is therefore an effective theoretical (σ-independent) PC, and the number of hydration water molecules around Na^+^ is a proxy for the σ-dependent score. Both experimental and theoretical spectra show a similar trend with negative applied potential/surface charge. Therefore, since the intensity in the theoretical spectra depends on the number of Na^+^ hydration water molecules, an intensity increase/decrease in the experiments correlates directly with an increase/decrease in the number of hydration waters around Na^+^ cations at the interface. Reading Fig. 2 *A* and *D* from top to bottom, we can hence assign the sharp intensity decrease detected in both the experimental and the theoretical spectra to the (partial) depletion of the hydration layer of Na^+^ at the interface, happening already at the smallest applied potentials/surface charges. The fact that the ΔA(ν; V_i_) maximum at ∼250 cm^−1^ in Fig. 2*A* is absent in the spectral bands of the solvated Na^+^ in Fig. 2*D* suggests that this feature does not arise from the Na^+^ hydration shell. Since this feature appears in the same PC as the bands < 200 cm^−1^, the underlying phenomenon must share the same voltage dependence as the Na^+^ rattling mode such that the PCA is not able to disentangle the two components. We propose that the ∼250-cm^−1^ band might be associated with changes in the interfacial water network upon changing the negative bias potential. Applying negative bias potentials induces adlayer disordering (13), which in turn leads to an intensity decrease around 250 cm^−1^ and an increase around 350 cm^−1^ (outside the observed range), compared to bulk water (see *SI Appendix*, Fig. S4). Our simulations show that Na^+^ hydration and interfacial water network disordering have a similar dependence upon negative charging of the Au surface (see Fig. 5 *A* and *C*), and thus we are unable to separate both by PCA.

Intriguingly, a very different behavior upon increase of the surface charge is observed for positive potentials. Supporting the experimental inference already discussed, the experimental spectral component in Fig. 2*B* is assigned to hydrated Cl^−^ after comparison with the theoretical spectra in Fig. 2*E*. Analogously to the cationic case for negative bias potentials, the spectra in Fig. 2*E* are obtained by multiplying the theoretical spectrum of bulk solvated Cl^−^ (39) by the σ-dependent change in the number of hydration water molecules in the first solvation shell of Cl^−^ in the EDL MD simulations versus bulk. Therefore, any change in intensity for this component can be associated with a change in the number of hydration waters solvating the anions at the interface. The spectral intensity is virtually constant in a large window of bias potentials/surface charges, implying that the Cl^−^ coordination shell is almost unaffected, while the stripping away of the anions occurs only at the most positive values, when the intensity starts to decrease in both experimental and simulated spectra. In the experiments (Fig. 2*B*) the onset of this decrease is at ΔV > 140 mV.

Concomitantly, a second component is observed for positive potentials (Fig. 2*C*) and assigned to the water network at the interface (see also *SI Appendix*, Fig. S5 for comparison with the THz absorption spectrum of bulk water). Strikingly, the center frequency of the underlying peak (147 ± 5 cm*^−^*^1^; *SI Appendix*, Fig. S6) resembles the hydration water band observed experimentally and theoretically in the hydration shell around hydrophobic alcohol chains (42, 43). This band arises from a wrapped HB interfacial–water network (43, 44), reminiscent of the planar 2D-HB network formed at the hydrophobic air–water interface (45) and at other planar interfaces such as graphene–water (46–49), where interfacial water HBs preferentially orient parallel to the surface. A similar in-plane interfacial water orientation has been reported for Au–water interfaces (21, 33). Importantly, this band is systematically red-shifted with respect to the 196-cm*^−^*^1^ band observed for bulk water (43, 44). We base this assignment on the comparison with the spectra in Fig. 2*F*, which show the experimental spectrum of the HB stretching mode of water molecules hydrating a hydrophobic surface (41), weighted by the variation of the number of HBs within the 2D-HB network as a function of σ.

The initial increase of the relative partial amplitudes for ΔV ≤ 80 mV in Fig. 2*C* is therefore attributed to a growth of the interfacial water network, i.e., to an increase in the number of HBs formed parallel to the Au surface in the interfacial layer. The position of the interfacial OH band (which depends on the strength of the HBs) does not change with potential. By contrast, the decrease for higher ΔV values reflects a partial breaking of the interfacial water structure. A comparison of the trends in Fig. 2*B* and Fig. 2*C* reveals that, at positive potentials, the spectral signature of the stripping off of the Cl^−^ hydration shell starts to be detected (Fig. 2*B*) only at potential values where the interfacial water network is partially broken (Fig. 2*C*).

Merging all the experimental results, we can infer that, for low positive potential values, the anions keep their full hydration layer, and the interfacial HB network is strengthened (i.e., a growth in the number of HBs), while cations directly lose part of their hydration shell already at the lowest negative potential in our experimental window. This goes beyond predictions based on continuous models, such as the GCS theory, which describes the ions’ accumulation at the interface in terms of the electrostatic interactions between the ions and the surface, without accounting for the specific ion–water and water–water interactions at the interface. At high positive/negative potentials, electrostatics become dominant and the textbook electrochemical picture from the GCS theory is restored: The hydration shells of both Cl^−^ and Na^+^ are depleted. Moreover, the asymmetry in the EDL formation at low positive and negative potential also contrasts with what is described in a number of electrochemical models, in which Na^+^ is expected to be more anchored to its hydration waters due to its higher charge density compared to Cl^−^ (48). Indeed, Na^+^ and Cl^−^ interact with different strength with their hydration water, and the free energy cost to remove one hydration water from the hydration layer of Na^+^ is higher than for Cl^−^, as confirmed by the potential of mean force derived from the MD simulations (see *SI Appendix*, section S12).

In order to confirm and rationalize the asymmetric stripping away of the Cl^−^ and Na^+^ hydration shells at the molecular level, we use a combination of classical MD simulations and ab initio calculations that naturally incorporate the potential of zero charge (PZC) as their internal reference. While the (static) ab initio calculations allow us to properly account for polarization and charge-transfer effects as a function of the applied voltage, the classical MD simulations allow us to simulate the low ionic concentrations used in the experiments and to perform a systematic investigation of the effects of surface charging, which are currently unfeasible by ab initio MD simulations with explicit solvent and applied bias. Therefore, these calculations allow us to investigate whether the asymmetric behavior observed experimentally still holds true when either the applied bias (ab initio) or the surface charge (classical MD) is varied symmetrically around the Au PZC.

**Fig. 3.**
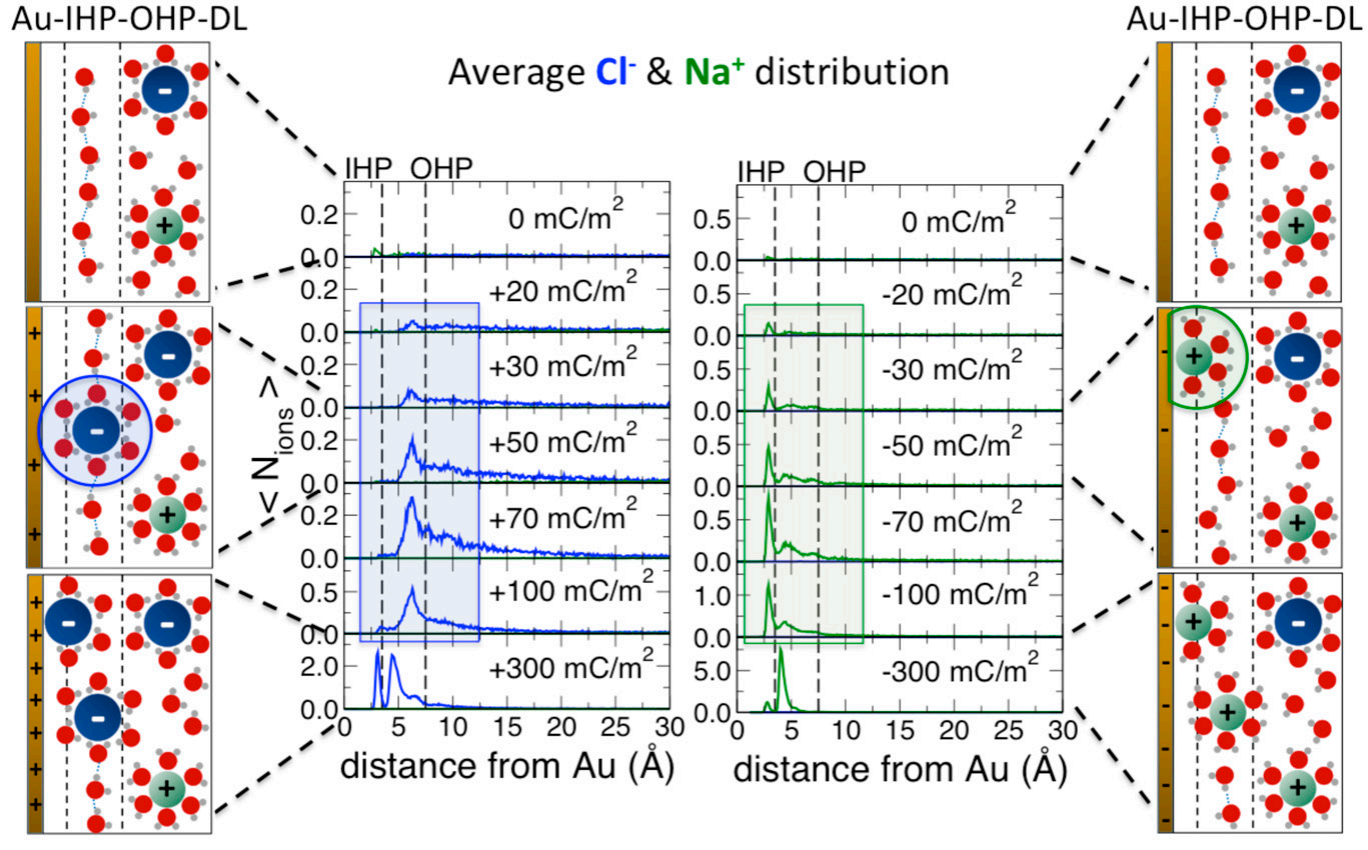
Microscopic view of the double-layer formation at the Au electrode. Average number of ions (Cl^−^ in blue, Na^+^ in green) as a function of the distance from a positively (*Left*) and negatively (*Right*) charged Au surface. Dashed lines mark the borders of the IHP and OHP. (*Left* and *Right*) Six key scenarios for ion distributions at zero, low, and high surface charge values (from top to bottom).

While the comparison with experimental results has been discussed earlier in the text, Fig. 3 displays the resulting molecular picture of the fundamental microscopic electrochemical processes derived from the classical MD simulations. We report the average ion distribution as a function of the vertical distance from the Au surface for all investigated positive (Fig. 3, *Left*) and negative (Fig. 3, *Right*) σ values. Both ion profiles show two peaks centered around 3 and 5 Å, corresponding to the inner and outer Helmholtz planes (IHP/OHP), respectively. The definition of the two planes is based on the distinct average coordination number of the ions. The density peak associated with the OHP identifies ions fully solvated within the water 2D-HB network located between 3 and 7 Å from the Au surface (see also *SI Appendix*, sections S10 to S12), where they maintain their hydration layer intact, i.e., same as in the bulk. By contrast, both anions and cations in the IHP directly face the bare Au surface and lose an average of one hydration water molecule. The change in the coordination of both ions with respect to the surface charge can hence be solely attributed to the way Cl^−^/Na^+^ populate the IHP.

As shown in Fig. 3, Cl^−^ preferentially stays in the OHP for low and intermediate positive σ and only migrates from the OHP to the IHP for the highest simulated voltage (i.e., surface charge σ >100 mC/m^2^) with a corresponding partial stripping of its hydration shell. In contrast, Na^+^ ions immediately populate the IHP at the lowest surface charge (σ = −20 mC/m^2^) and start to occupy the OHP only when the IHP is saturated at around σ = −70 mC/m^2^. When chloride ions accumulate in the OHP, we observe an increase in the number of interfacial water molecules oriented with both O–H groups parallel to the Au surface, leading to an increase in the number of parallel HBs forming the 2D-HB network (see also Fig. 5*D* and *SI Appendix*, Fig. S12). This growth is lost once the anions move from the OHP to the IHP. Water molecules are then forced to align with respect to the high electrostatic field generated by the charged surface. Due to the reorientation, the number of in-plane HBs formed between interfacial waters decreases, thereby weakening the 2D-HB network. As long as the 2D-HB network is still present at the interface (σ ≤ 100 mC/m^2^), anion location in the OHP is therefore favored by both ion–water and water–water interactions. In contrast, the 2D-HB network growth is not observed for negatively charged surfaces (see Fig. 5*C*) for which interfacial water molecules orientation progressively changes from in-plane to pointing toward the surface. Thus, insertion of Na^+^ into the IHP starts immediately upon application of a negative voltage as observed in the experiments and simulations.

The reported changes in interfacial water structure are in good agreement with a recent combined experimental and ab initio study (21), concluding that the topmost interfacial layer at the Au–water interface is mostly oriented parallel to the surface at 0 V. When applying an increasingly negative potential, water was found to reorient first with one OH, and finally with both OH pointing toward the surface (*SI Appendix*, Fig. S12). Such behavior has been also described by MD simulations in ref. 13, revealing that under a negative bias the reorientation of water molecules with H atoms toward the Au surface disrupts the 2D-HB network in the interfacial layer. Furthermore, our results on the in-plane orientation of water that persists longer under positive surface charging supports the idea of an asymmetric reorientation of water at positive/negative electrodes as described in refs. 13 and 49. The result provided here is that asymmetry in the response of the water adlayer to an applied voltage causes anions and cations to migrate differently when positive/negative potentials are applied.

**Fig. 4.**
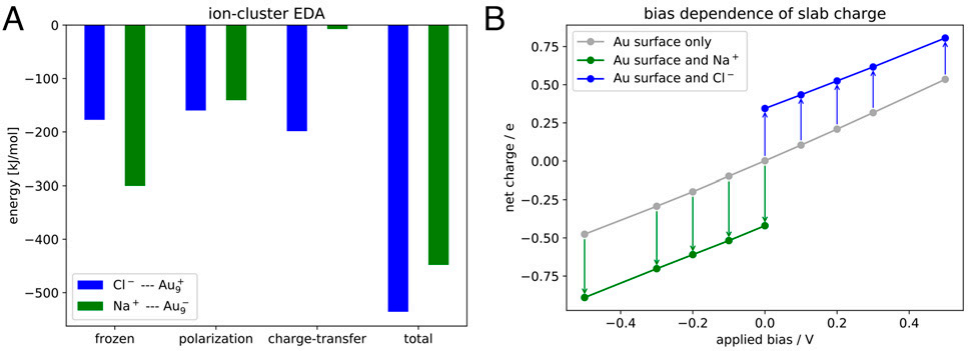
Results of electronic structure calculations. (*A*) Comparison of the different contributions to the interaction energy of the Na^+^ (green) and Cl^−^ (blue) ion with a negatively and positively charged Au_9_ cluster model, respectively, as calculated with ωB97X-V/def2-TZVPD. While the polarization term is of equal magnitude, charge transfer is completely absent in case of Na^+^, whereas it contributes to the interaction energy for the Cl^−^ case. (*B*) The net charge of a grand-canonical periodic slab model with varying bias potential referenced against the computed PZC. The green (blue) data points show the corresponding decrease (increase) of the net charge upon Na^+^ (Cl^−^) binding. The change of this polarization effect is negligible in the experimental bias range (−0.15 to 0.2 V) as indicated by the constant length of the colored arrows.

However, these calculations do not account for polarization and chemical changes induced by the ions and the applied bias. Energy decomposition analysis of charged Au cluster models (Fig. 4*A* and *SI Appendix*, section S16) demonstrates that polarization plays a role but is independent of the charge. These calculations also reveal charge transfer from the chloride ion to the Au cluster that is completely absent in the Na^+^–Au interaction. However, the fact that these effects are independent of cluster charge rules them out as the source of the different behavior of Na^+^ and Cl^−^ at the electrochemical double layer. Grand-canonical (in electrons) periodic slab calculations on an Au(100) surface additionally confirm that the polarization of the electrode as measured by the change of number of electrons present in the metal slab is largely independent of the applied bias (Fig. 4*B* and *SI Appendix*, section S17) in the experimentally accessed range. In addition, a microsolvation analysis (*SI Appendix*, section S18) demonstrates that Cl^−^ prefers a surface-like asymmetric microsolvation pattern as is present in the hydrophobic OHP (cf. ref. 33), whereas Na^+^ prefers to be symmetrically solvated, which can be achieved at the IHP by replacing one or two water molecules with negatively charged Au atoms. All in all, the quantum-chemical calculations support the results obtained from experiment and the classical MD simulations and hint that the deficiencies of a classical fixed charge model do not lead to biased conclusions in the current study.

In conclusion, by a THz spectroelectrochemical approach combined with MD simulations we have directly probed the solvation/desolvation processes at the Au surface under *operando* electrochemical conditions. Our results have dissected the stripping away of the hydration shells of the electrolyte ions as well as the associated changes in the interfacial water HB network at the microscopic level. Our approach can now be used to investigate the crucial role of water in mediating other interfacial processes at metal as well as semiconductor/electrolyte interfaces. This view will impact the understanding and optimization of electrochemical processes for technological applications.

## Methods

### THz Spectroelectrochemical Measurement.

THz-far IR absorption spectra of 10 mM NaCl (Sigma-Aldrich, purity ≥98%) were recorded in the frequency range 50 to 350 cm^−1^ with 2-cm^−1^ resolution. We employed a vacuum-evacuated (10^−5^ mbar) Fourier transform IR spectrometer (IFS 125; Bruker) with an external 4.2 K He-cooled bolometer (Infrared Laboratories) for the detection of the synchrotron light (at the beamline AILES, Soleil). The source has a flux of 5·10^13^ photons per s per 0.1% bandwidth at 100 cm^−1^. Each single spectrum is the average of 128 scans recorded at 40 kHz at 25 °C. To apply constant potential, we used a potentiostat (PalmSens 4; PalmSens BV) and connected the counterelectron (CE) and reference electrode (RE) leads of the potentiostat to the CE and its working electrode (WE) lead to the WE (2-electrode setup).

From each spectrum at the potential V_i,_ the previously recorded spectrum at the potential V_ref_ is subtracted. The difference spectra are then decomposed by PCA into independent spectral components (PCs), based on their distinct response to the potential increase/decrease. The changes in the total spectrum are then attributed to voltage-dependent changes of the respective partial spectra. One spectral component for the negative potential series and two spectral components for the positive one are sufficient to describe the voltage dependent changes in the spectral dataset.

### MD Simulations.

Classical simulations were performed using the LAMMPS (50) code to simulate 17 aqueous solutions (17,486 water molecules) of 20 mM NaCl confined between parallel charged Au(100) walls with imposed 298 K constant temperature to water and ions all along the simulation. The simulated surface charge values are 0, ±20, ±30, ±50, ±70, ±100, ±170, ±240, and ±300 mC/m^2^. Since no ion-pairing interactions are detected in the MD simulations with 20 mM NaCl (<0.01% probability to have an ion pair), no differences are reasonably expected between 10 mM (experimental concentration) and 20 mM concentrations, which are relevant to the conclusions of this work. The constant charge is imposed on the topmost atomic layer. The water + NaCl system was described using the force field developed by Kann and Skinner (51), based on the TIP4P/2005 model of water and employing rescaled (by 0.85) charges for ions. Charge rescaling compensates for the underestimated permittivity of TIP4P/2005 water and effectively describes ion polarizability and charge delocalization of solvated ions (51). We systematically rescaled the surface charge with the same ratio as the one applied to ions. The interactions between Au atoms and liquid atoms were treated using the Lennard-Jones parameters introduced by Heinz et al. (52) and Lorentz–Berthelot mixing rules. Three-dimensional periodic boundary conditions were applied with lateral dimensions of 56.213 Å and counterions were added to the system to ensure electroneutrality. To impose a pressure of 1 atm, we used the top wall as a piston until an equilibrium height was reached, and we fixed the top wall height at its equilibrium position for the rest of the equilibration and for the production runs. The same simulation protocol with an equilibration run of 36 ns (with a timestep of 1 fs) followed by a production run of 36 ns (with a timestep of 2 fs) has been systematically followed. The total simulation time, including the equilibration period where the upper solid surface is used as a piston, is about 90 to 100 ns for each simulation. We carefully checked that at the end of the equilibration time ions (and water) were correctly distributed in the simulation box with correct density profiles.

**Fig. 5.**
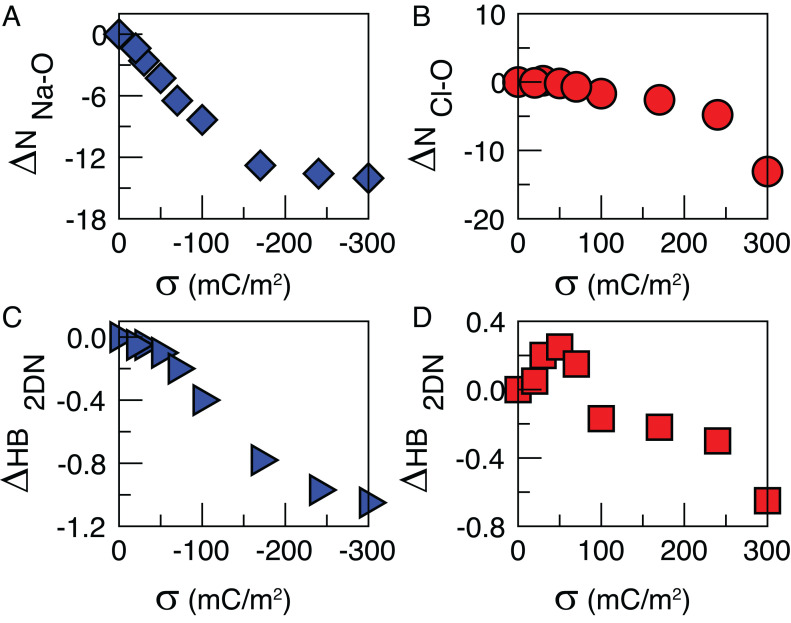
Theoretical scores used to calculate the spectra shown in Fig. 2 *D*–*F*. (*A*) Differences in the total Na-O coordination numbers with respect to negative surface charging. (*B*) Differences in the total Cl-O coordination numbers with respect to positive surface charging. (*C*) Average number of HBs per molecule formed between water molecules in the topmost interfacial layer and oriented parallel to the surface for negative surface charging. (*D*) Average number of HBs per molecule formed between water molecules in the topmost interfacial layer and oriented parallel to the surface for negative surface charging.

The differences in the total Cl-O and Na-O coordination numbers [ΔN_Cl-O_(σ) and ΔN_Na-O_(σ), respectively] with respect to the positive and negative surface charging, used as “theoretical scores” to calculate the spectra in Fig. 2 *D* and *E* are derived as follows:

$$\Delta {\text{N}}_{\text{ion}}{\left(\sigma \right)}_{=}{\text{N}}_{\text{ion}}{}^{\text{int}}\left(\sigma \right)({\text{coord}}_{\text{ion}-\text{O}}{}^{\text{int}}\left(\sigma \right)-{\text{coord}}_{\text{ion}-\text{O}}{}^{\text{bulk}}),$$

where N_ion_^int^ (σ) is the number of Cl/Na in the interfacial layer (i.e., in the IHP+OHP), coord_ion-O_^int^(σ) is the average Cl/Na coordination number in the interfacial layer at a given σ value, and coord_ion-O_^bulk^ is the average Cl/Na coordination in bulk. The differences in the total Cl-O and Na-O coordination numbers (shown in Fig. 5 *A* and *B*) represent the variation in the coordination of all ions at the interface with respect to their coordination in the bulk. In order to obtain the theoretical spectra in Fig. 2 *D* and *E*, the theoretical spectra of solvated Na^+^ and Cl^−^ from ref. 39 are scaled by these scores. The theoretical spectra of solvated Na^+^ and Cl^−^ are obtained as the sum of self- and cross-correlation terms between the ion, the water molecules in their first solvation shell, and the ones in the second shell. The theoretical scores used to calculate the spectra in Fig. 2*F* are related to the strength of the 2D-HB network, measured as the average number of HBs formed between water molecules in the topmost interfacial layer and oriented parallel to the surface (HBs_2DN_/molecule). The results obtained for both positive and negative surface charge values are plotted in Fig. 5 *C* and *D*. Water–water HBs are defined using a mixed distance-angle criterion, with an O–O distance cutoff of 3.2 Å and the O–H··· O angle in the range [140,220]°. Different HB criteria (e.g., O–O distance cutoff of 3.5 Å and O–H··· O angle in the range [150,210]°) lead to the same trends as reported in Fig. 5. Moreover, the changes in the water network upon negative surface charging (Fig. 5*C*) show a trend similar to ΔN_Na-o_ (Fig. 5*A*). Therefore, PCA is not able to separate the possible spectral contribution of the change in the interfacial water network from the Na^+^ hydration. This can explain why a water-related spectral component is not observed in the experiments for the negative potential series.

### Electronic Structure Calculations.

All density functional calculations for the cluster models were carried out with the Q-Chem electronic structure package (53). We applied the RPBE functional (54) for consistency with the periodic slab calculations and the ωB97X-V functional (55) for comparison. The density functional theory calculations for the periodic slab models were carried out on the Au(100) surface using the Vienna Ab Initio Simulation Package (56, 57). The surface unit cell (periodically extending in the *x* and *y* directions) was designed as four layers of eight Au atoms, with a lattice constant of 4.0782 Å and 20 Å spacing (in the *z* direction) between two images. A *k*-point sampling of the Brillouin zone was achieved using the 4 × 4 × 1 Monkhorst−Pack mesh. The implicit aqueous solvent and the electrolyte were implemented with a dielectric constant $\varepsilon_{r}=78.4\;$ and a Debye–Hückel length $\lambda_{b}=9.61$ Å. More details are given in *SI Appendix*.

## Supplementary Material

Supplementary File

## Data Availability

Input files and raw data used for the figures have been deposited in Zenodo (https://zenodo.org/record/5545880#.YWXCzBxOlPY). All other study data are included in the article and/or *SI Appendix*.
