# Supplementary material for: Stripping away ion hydration shells in electrical double-layer formation: Water networks matter
Source: Proc Natl Acad Sci U S A. 2021 Nov 15;118(47):e2108568118. doi: 10.1073/pnas.2108568118 (PMC8617503; doi:10.1073/pnas.2108568118)
Supplement: Supplementary File [file pnas.2108568118.sapp.pdf]

List of supplementary materials:

Sections S1-S6: Experimental materials, methods and data analysis details

Section S7: Raw data

Section S8: Reference electrode in the experiments

Section S9: Experimental THz absorption spectra of NaCl solutions

Sections S10-S13: Classical molecular dynamics simulations setup and supplementary analyses

Sections S14-S18: Ab-initio calculations setup and supplementary analyses

Figures S1-S22

Tables S1-S2

References: 58-72

Supplementary Materials for

Stripping away ion hydration shells in electrical double layer formation: water networks matter

Serena R. Alfarano[1,a], Simone Pezzotti[1,2,a], Christopher J. Stein[7,8], Zhou Lin[6,7], Federico Sebastiani[1], Sarah Funke[1], Claudius Hoberg[1], Inga Kolling[1], Chun Yu Ma[1], Katja Mauelshagen[1], Thorsten Ockelmann[1], Gerhard Schwaab[1], Li Fu[3], Jean-Blaise Brubach[4], Pascale Roy[4], Martin Head-Gordon[6,7,*], Kristina Tschulik[5,*], Marie-Pierre Gaigeot[2,*], Martina Havenith[1,*]

[1] Lehrstuhl für Physikalische Chemie II, Ruhr–Universität Bochum, 44780 Bochum, Germany

[2] LAMBE UMR8587, Université d'Evry val d'Essonne, CNRS, CEA, Université Paris-Saclay, 91025, Evry, France

[3] Univ Lyon, Univ Claude Bernard Lyon 1, CNRS, Institut Lumière Matière, F-69622 Villeurbanne, France

[4] Synchrotron SOLEIL, AILES beamline, L'Orme des Merisiers, Saint Aubin, BP 48, 91192 Gif sur Yvette Cedex, France

[5] Micro- & Nano-Electrochemistry, Center for Electrochemical Sciences (CES), Ruhr-Universität Bochum, ZEMOS 1.45, Universitätsstr. 150, 44801, Bochum, Germany

[6] Chemical Sciences Division, Lawrence Berkeley National Laboratory, Berkeley, California 94720, United States

[7] Department of Chemistry, University of California, Berkeley, Berkeley, California 94720, United States

[8] Theoretische Physik, Universität Duisburg-Essen and CENIDE, 47048 Duisburg, Germany

[a] These authors contributed equally: S.R. Alfarano, S. Pezzotti

[*]email: mhg@cchem.berkeley.edu, kristina.tschulik@rub.de; mgaigeot@univ-evry.fr;

martina.havenith@rub.de

S1. Experimental materials and methods

Electrochemistry measurements

We adopted the electrochemical cell developed at AILES [41], consisting of two wedge THz transparent diamond windows, a 4-6 µm thick Au grid placed in between the diamonds as the working electrode (WE) and a thin platinum foil deposited at the window periphery as counter electrode (CE). We did not employ a reference electrode (RE) during the spectra acquisition to prevent concentration alterations of the sample solution. THz absorption measurements were carried out simultaneously with chronoamperometric measurements. To apply constant potential, we used a potentiostat (PalmSens 4, PalmSens BV), and connected the CE and RE leads of the potentiostat to the CE and the WE lead to the WE (2-electrode setup). The assembly of the cell took place under argon atmosphere to avoid oxygen contamination.

THz spectroscopy measurement

We recorded THz far infrared (THz/FIR) absorption spectra of 10 mM aqueous NaCl (Sigma-Aldrich, purity ≥ 98%) in the frequency range 50-350 cm^{-1} with 2 cm^{-1} resolution as a function of a constant external bias potential. The NaCl concentration is intentionally kept low to maximize the volume of the electrical double layer. We used a vacuum-evacuated (10^{-5} mbar) Fourier transform infrared spectrometer (FTIR, IFS 125, Bruker) with an external 4.2 K He-cooled bolometer (Infrared Laboratories, Inc., Tucson, USA) for the light detection and the synchrotron light (at the beamline AILES, Soleil) as a source of radiation. The source has a flux of 5.10^{13} photons/s/0.1% bandwidth at 100 cm^{-1}. Each single spectrum is the average of 128 scans recorded at 40 kHz at a constant temperature of 25 °C. For each applied bias potential V_i, we collected 20 consecutive spectra at identical conditions. We started the acquisition of the first spectrum of the series only after achieving current equilibration. Each spectrum at constant bias potential V_i and is followed by a new measurement of the open circuit potential (OCP) reference spectrum at V_{ref}. We acquired these reference spectra after each spectrum at a potential V_i to confirm that the background was constant during the total acquisition time. The data for the

reference spectra are reported in section S4.

Data analysis

From the Lambert-Beer' law the absorbance A at a certain potential V_i is expressed as:

$$A(\nu;V_i) = -(\ln I(\nu;V_i) - \ln I(\nu;V_{max})) \quad (1)$$

where $I(\nu;V_i)$ and $I(\nu;V_{max})$ are the transmitted intensities at the applied potential V_i and at the maximum applied potential for each series, V_{max}, respectively.

In the main text we discuss two absorption spectra datasets, one for positive applied bias potential positive and one for negative applied potential measured with respect to the open circuit reference potential V_{ref}. The positive potential series covers a potential range between $V_{ref,pos}$ = 0.03V and $V_{max,pos}=V_{ref,pos}$ +0.2 V in 20 mV steps (V_i), while the negative potential series covers a potential range between $V_{ref,neg}$ = 0.07V and $V_{max,neg}=V_{ref,neg}$ -0.15 V with spectra taken at $V_i = V_{ref,neg}$ - [2 mV, 4 mV, 6 mV, 8 mV, 10 mV, 20 mV, 30mV, 50 mV, 100mV, 150 mV]. Each spectrum acquisition at a potential V_i was followed by the measurement of a spectrum at the reference potential V_{ref} to confirm constant background.

We performed a principal component analysis (PCA) on the total set of $\Delta A(\nu;V_i)$ spectra for the positive and negative potential series in order to obtain an orthonormal basis set carrying the fundamental effect of the applied bias potential on the THz spectra in the region between 50-350 cm^{-1}. A rectangular matrix **M**, where each row contains a spectrum $\Delta A(\nu; V_i)$, is decomposed into its PCs via singular value decomposition (SVD). The SVD decomposes **M** into a triple product of the left eigenvectors, **U**, a diagonal matrix **X** of singular values that we refer to as the scores x_a, and the transpose of the matrix of right eigenvectors, **V**, such that $\mathbf{M}=\mathbf{U}\mathbf{X}\mathbf{V}^T$. The principal components, $(\mathbf{u}_a,\mathbf{v}_a^T)$ are the eigenvectors ordered according to their singular values (variances) such that the first principal component, PC 1, has the largest variance and they provide the spectral information. In the main text, we refer to the principal components as partial spectra. The scores, x_a, are the contribution of each principal component to the total spectrum as a function of the applied potential. More details are available in Ref. 38.

The scores x_a are shown in Figure S1 for both positive (two PCs) and negative applied potential series (only one PC). Their trend as a function of the applied potential agrees well with the theoretical scores derived from the MD simulations as a function of surface charge and reported in Figure 5 of the main text.

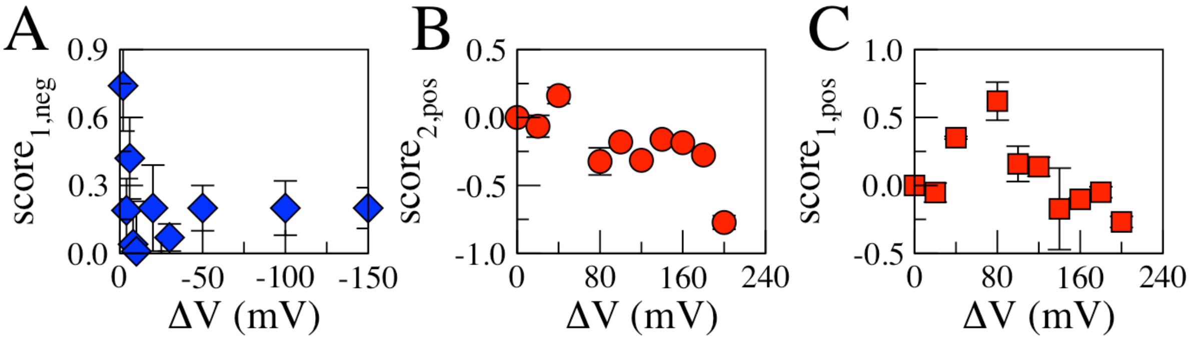

***Figure S1: (A)** Scores obtained from the first PC of the negative potential series. The first data point is at 2 mV. **(B)** Scores associated with the second PC from the positive potential series. **(C)** Scores associated with the first PC from the positive potential series. For some data points, the errors bars are very small and obscured behind the symbol.*

S2. Cyclic voltammogram of 10 mM NaCl

In order to identify a suitable potential range for the THz measurements, we measured cyclic voltammograms (CVs) of 10 mM NaCl in the range from -0.3 V to 0.3 V in 10 mV steps (see Figure S2). The acquisition conditions were the same as the ones described in section S1: the electrochemical cell is composed of a Au thin foil as a working electrode (WE), a Pt wire as counter electrode (CE) and we did not make use of a reference electrode (RE) to avoid contamination in the sample solution concentration. The CE and RE leads of the potentiostat are connected to the CE and its WE lead is connected to the WE (2-electrode setup). Our aim was to find a potential range in which no reactions (e.g. water splitting) take place to prevent the formation of bubbles that could compromise the spectral results.

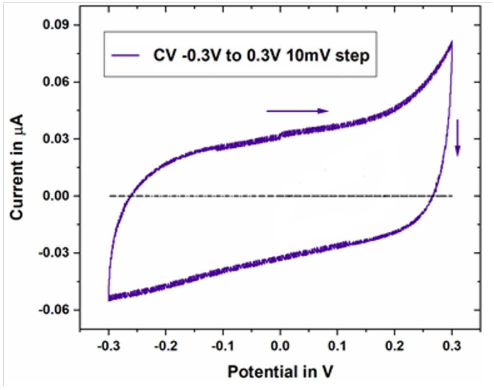

Figure S2: *CV of 10 mM NaCl in the range -0.3 V to 0.3 V in 10 mV steps.*

S3. THz spectra: Calculation of the effective probing volume

The Au grid (GoodFellow GmbH) used in the experiment as the working electrode was a 4 µm thick mesh, with square holes of side length 64 µm, spaced 12 µm apart, as shown in Figure S3, corresponding to an open area of 70%. We presume that the THz radiation passes through the holes of the grid. The volume of the hole is V_{hole} = 64 µm x 64 µm x 4 µm = 16384 µm^3. With 10 mM NaCl, the electrical double layer (EDL) thickness is expected to be roughly 3 nm (based on the Debye length, including the diffuse layer), so the EDL volume probed by the THz radiation in the open part of the mesh is V_{EDL} = (64 µm x 4 µm x 3 nm) x 4 = 3.072 µm^3, where the factor of 4 stems from the 4 walls of the hole at which the EDL is formed. In this way, the ratio between the two volumes is $V_{EDL}/V_{hole} = 2{\cdot}10^{-4}$. We associate this ratio of $2{\cdot}10^{-4}$ with the minimum variation between two measurements that we can detect in our experiment.

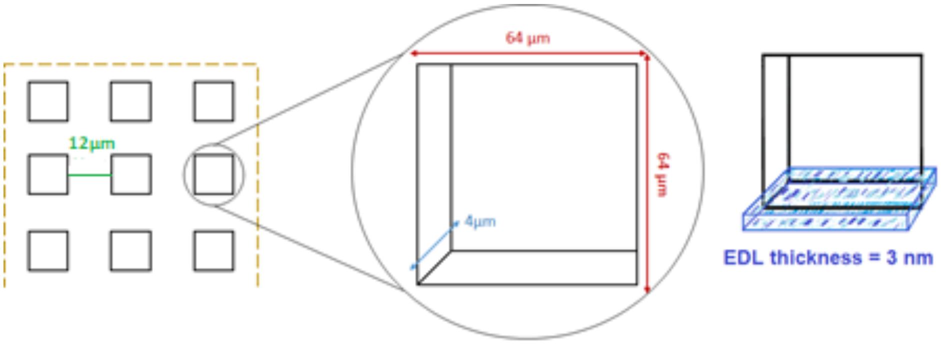

Figure S3: *Schematic representation of the Au mesh working electrode, where the squares are*

holes in the gold foil. On the right, there is a schematic diagram of the formation of the electrical double layer on a side of the hole.

S4: Reference spectra (background)

Positive potentials

In Figure S4-A we report the evolution of the background (BG) contribution to the reference spectra ($\Delta A_{BG}(\nu)^{j}$), calculating the difference between two subsequent measurements at V_{ref}:

$$\Delta A_{BG}(\nu)^{j} = \ln I(\nu; V_{ref}^{j+1}) - \ln I(\nu; V_{ref}^{j}) \qquad (2)$$

Figure S4-A shows that the background contribution is generally featureless although there are small variations due to factors such as changes in synchrotron power, temperature changes, etc. These systematic errors are canceled by referencing each spectrum to the corresponding BG to yield the results in Fig. 1 of the main paper (see also SI Sec. S7). A small apparent feature around 60 cm^{-1} is attributed to the synchrotron itself.

Negative potentials

Similar to the positive potential series, we report the evolution of the background contribution to the reference spectra ($\Delta A_{BG}(\nu)^{j}$) for the negative potential series in Figure S4-B. Since the background is constant within experimental uncertainty, and the spectra taken at the negative potentials shown in Fig. 2-A of the main text have been corrected for the background, we conclude that the band at around 250 cm^{-1} in Fig. 2-A in the main text cannot be attributed to background noise and is indeed a feature. We assume that this peak is due to disruption of the interfacial water network upon the application of the negative bias potential. To arrive at this conclusion, we have compared the calculated (*cfr* Ref. 43) spectrum of the air-water interface shown in Figure S4-C, with the THz absorption spectrum in Figure 2-A. The features in the high frequency region of the calculated spectrum (the valley at 250 cm^{-1} and the intensity increase at higher wavenumbers) are associated with the 2D-HB network. The latter is partially disrupted with increasing applied negative bias potential, leading to a progressive removal of the intensity at around 250 cm^{-1} in the experimental partial spectrum (Figure 2-A).

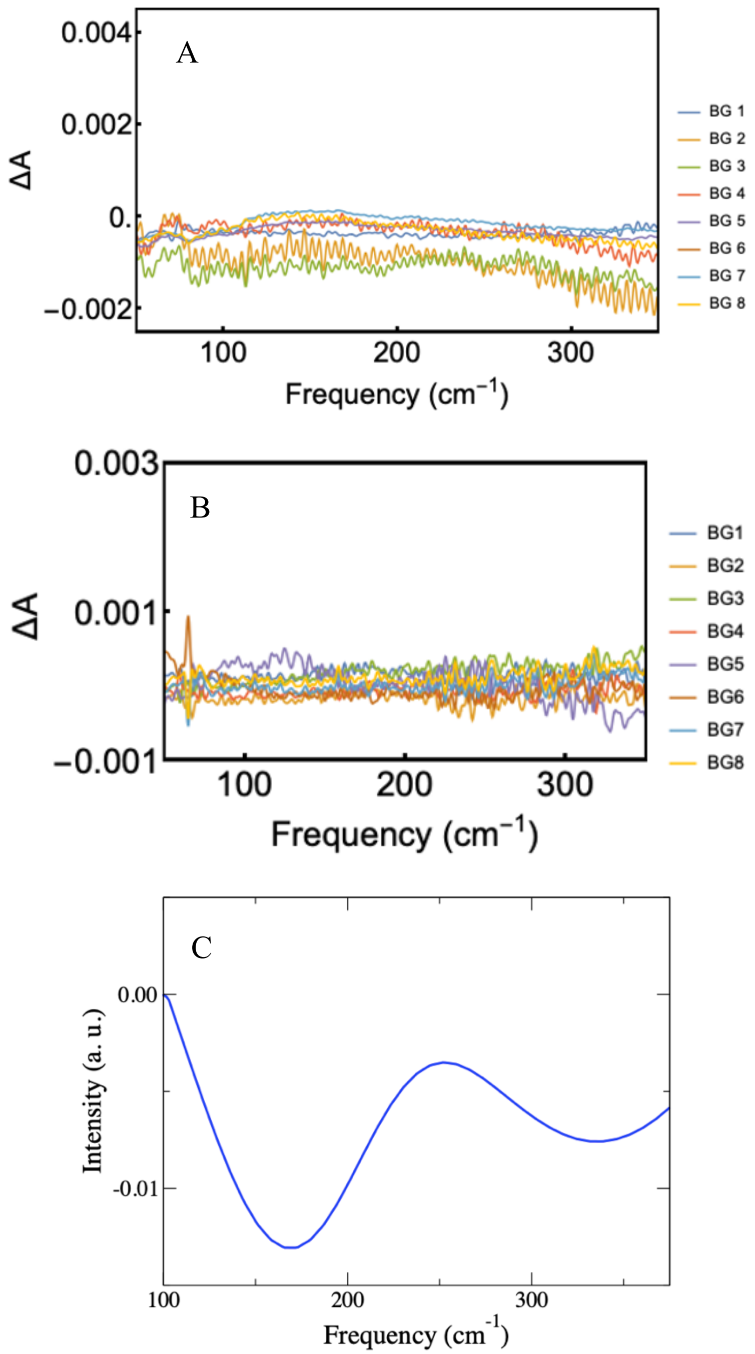

Figure S4: A) *Background contribution* (ΔA_{BG} (ν) ^{j}, see *Eq. 2) calculated for the set of 8 reference spectra for the positive potential series.* ***B)*** *Background contribution* (ΔA_{BG} (ν) ^{j}, see *Eq. 2) calculated for the set of 8 reference spectra for the negative potential series .* ***C)*** *THz absorption spectrum of the air-water interface corrected for bulk water, calculated by MD simulations in Ref. (43).*

S5. Comparison of the first partial spectrum at positive potential with pure water

As discussed in detail the manuscript, we associate the first PC of the positive potential series with pure water. In Figure S5 we compare the first partial spectrum (PC1, black curve) and the THz absorption spectrum of bulk water (blue curve). The similarities of these band profile confirm that this partial spectrum is related to pure water, but cannot be identified as bulk water. We hence (and because of the results of our theoretical calculations shown in Fig. 2-F of the main text) assign this partial spectrum to interfacial water.

We further note a low intensity feature at around 60 cm^{-1}, which is not observed in the bulk water spectrum. However, due to the sharpness of the band and its appearance also in the background spectra (see Figure S4-A), we attribute this feature to the characteristic of the synchrotron and a residual noise contribution.

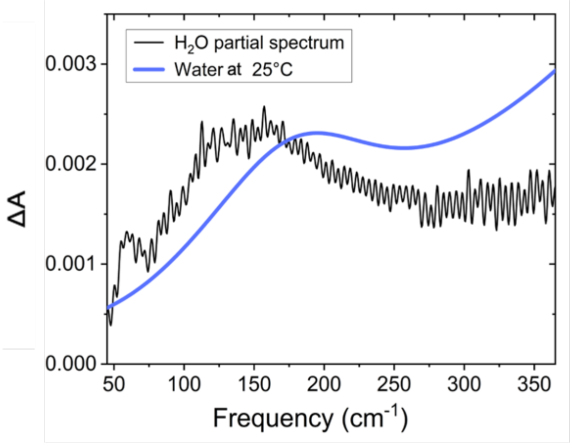

Figure S5: *Comparison between the first partial spectrum of the dataset for the positive potential series (black curve) and the absorption spectrum of bulk water at 25°C (blue curve).*

S6. THz spectra: fit of the first partial spectrum

Figure S6 shows a decomposition of a fit to the PC 1 of the positive potential series that was discussed in the previous section. We used the following fitting function f to decompose the partial spectrum into three distinct components:

$$f\,(\nu, c_{LF}, \nu_D, A_0, \Delta, c_{Lib}) = c_{LF}\,\frac{a\,e^{-\frac{\nu}{\nu_{c,0}}}}{\pi\left(\nu^2+\frac{\Delta^2(0)}{\pi^2}\right)}\nu^2 + \frac{A_0\Delta^2}{4\pi^3\left[\left(\nu_D{}^2+\left(\frac{\Delta}{2\pi}\right)^2-\nu^2\right)^2+4\left(\frac{\Delta}{2\pi}\right)^2\nu^2\right]}\nu^2 +$$

$$c_{Lib}F_{Lib,DHO}\,(\nu, \tilde{\nu}_D, \tilde{A}_0, \widetilde{\Delta}) \qquad (3)$$

The first addend describes the low frequency part of the spectrum (denoted "LF" in Figure S6), using a Debye term scaled by c_{LF}, the second term (denoted "Peak @ 147 cm^{-1}" in Figure S6) corresponds to a damped harmonic oscillator (DHO), while the third term (denoted "Libration" in Figure S6) describes the absorption in the higher frequency part of the spectrum (ν >300 cm^{-1}) and is approximated by the librational band of bulk water at 25°C with two damped harmonic oscillators multiplied by a fitting parameter c_{Lib}. Furthermore, ν denotes to the frequency, ν_D is the center frequency of the band, A_0 is the amplitude and Δ is the width of the band, respectively. The corresponding unperturbed center frequency ν_0 of the oscillator can be calculated from: $\nu_0 = \sqrt{\nu_D{}^2 + \left(\frac{\Delta}{2\pi}\right)^2}$

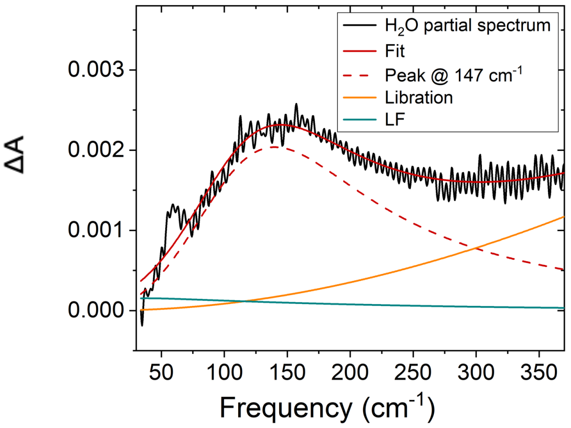

Figure S6: *Fit (solid red curve) of the positive potential series partial spectrum (black curve) assigned to interfacial water and its decomposition into three components (see Eq. (3))..*

Fit parameters:

**C_{Lib}**	0.000046 ± 0.00002
**C_{LF}**	0.000044 ± 0.00001
**A_0 [Intensity, arb.unit]**	0.0256 ± 0.0003
**Δ [cm^{-1}]**	577 ± 20
**ν_D [cm^{-1}]**	114 ± 3

Table S1: *Parameters of a fit with Eq. (3) to the first principal component of the positive potential series.*

From the fitted parameters in Table S1, we obtain the unperturbed center frequency of the 2D-HB network as ν_0 = 147± 5 cm^{-1}.

S7: Additional and alternative data analysis

Experiments: Positive potential series

Each spectrum is the average of 20 consecutive measurements. Figure S7-A shows the calculated $\Delta A(\nu;V_i)$ as calculated from Eq. (1). We analyzed these spectra (Figure S7-A) via PCA. From this PCA (see section S1), two main spectral components are found to be sufficient to fully describe the evolution of the dataset with the positive applied potential. As a complementary alternative analysis motivated by this finding, the spectra in Figure S7-A have been fitted with two damped harmonic oscillators, assigned at the interfacial water network band (147 cm^{-1}, see previous section) and to the Cl^- hydration layer (~ 200 cm^{-1}), respectively. The results are displayed in Figure S7-B.

In Figure S7-C, the amplitudes of the two bands are shown as a function of the applied positive potential. The voltage dependence resembles that of the scores obtained from the PCA (see Figure S1). However, we chose PCA in our analysis in the main text, since it is a) model-independent and b) able to describe non-Gaussian or non-Debye like lineshapes (including negative partial contributions to the overall absorption).

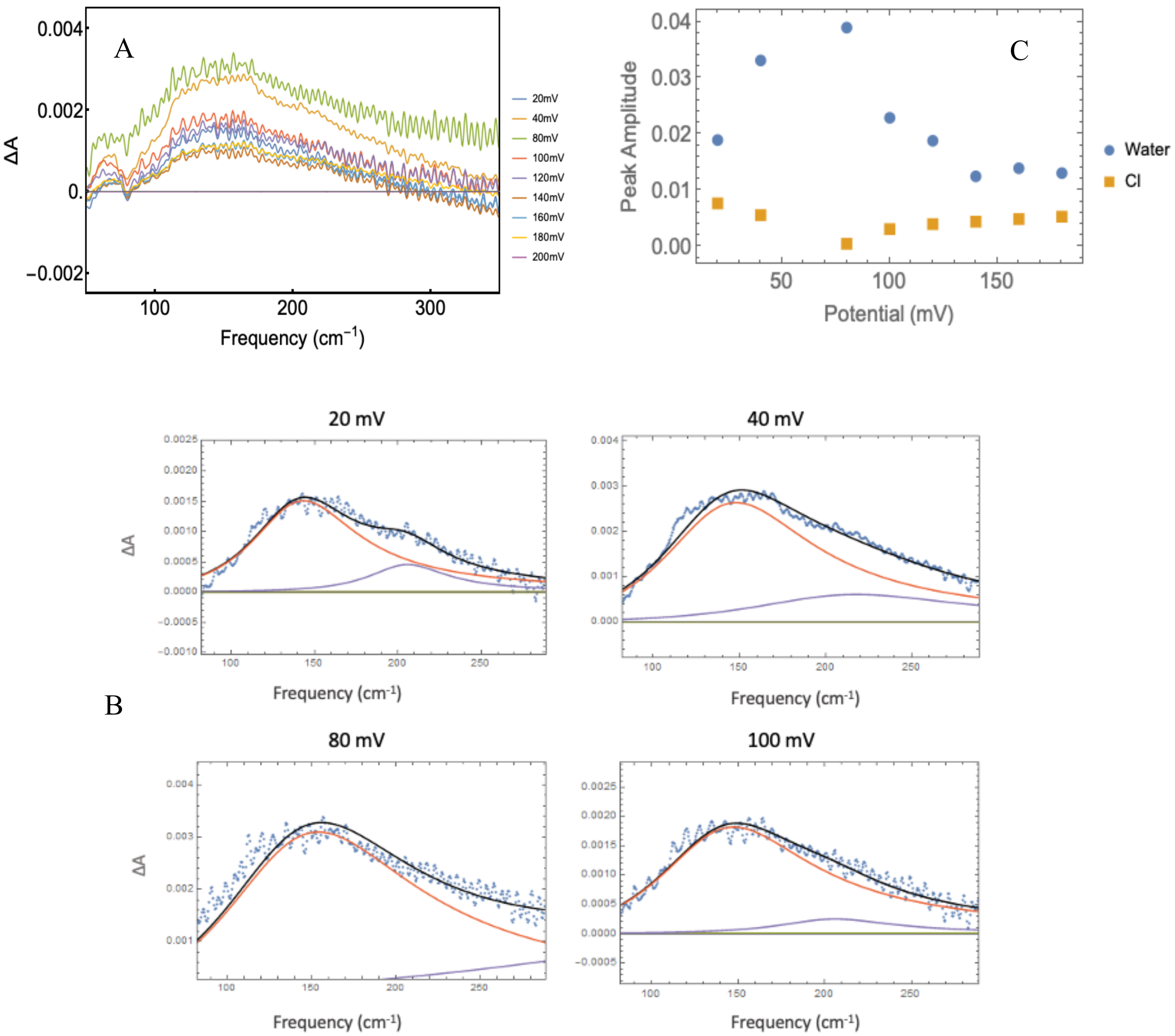

A
B
C
ΔA
Frequency (cm⁻¹)
20mV
40mV
80mV
100mV
120mV
140mV
160mV
180mV
200mV
Peak Amplitude
Potential (mV)
Water
Cl
20 mV
40 mV
80 mV
100 mV

B

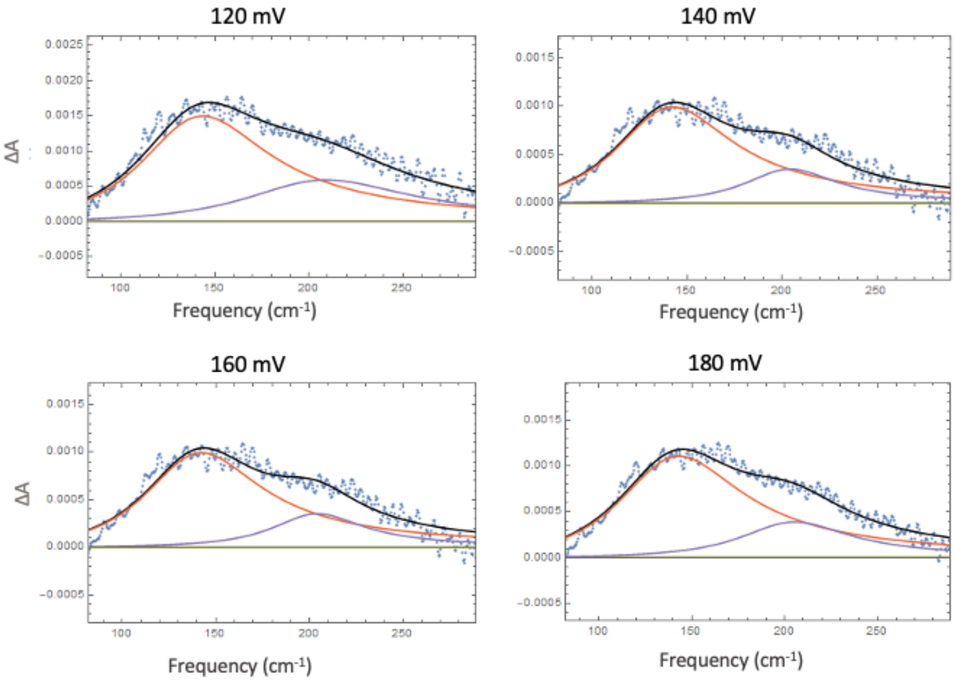

Figure S7: *(**A**)* $\Delta A(\nu;V_i)$ *calculated from Eq. (1) for the positive potential series. (**B**) Dissection with two damped harmonic oscillators (DHO) of the spectra in panel (A). (**C**) Amplitudes of the fitted bands in panel (B) as a function of the positive applied potential.*

Experiments: Negative potential series

For the negative potential series, the results presented in the main text and in the SI are derived from the average of two datasets acquired under identical conditions. Each spectrum of each of the two datasets is the average of 20 consecutive measurements. Figure S8 shows $\Delta A(\nu;V_i)$ calculated from Eq. (1) and averaged over the two datasets. The matrix **M** that is decomposed by the PCA is constructed from the corresponding spectra in Figure S8-A. The first PC is shown in Fig. 2 of the main text. Figure S8-B reports the second PC. It is featureless and has zero intensity within the experimental uncertainty over the whole frequency range probed confirming that the first PC contains all applied bias dependent spectral changes.

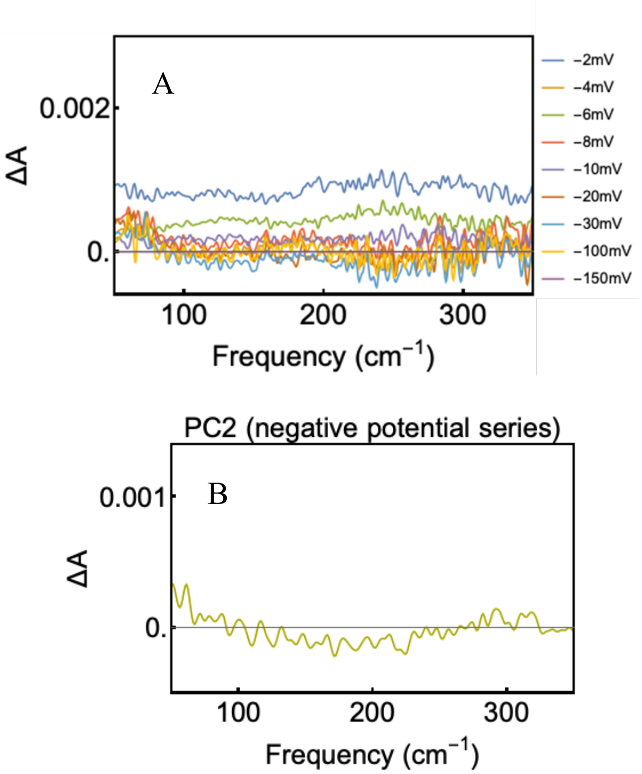

Figure S8: A) ΔA(v;V_i) *calculated from Eq. (1) for the negative potential series.*

B) *Experimental PC 2 derived for the negative potential series.*

S8. Use of the reference electrode

In order to reference the bias potentials to a Ag/AgCl reference electrode (RE), we measured the open circuit potential with a Ag/AgCl, 3 M KCl RE, in two distinct measurements before spectral acquisition at positive and negative biases. The results obtained were +0.03 V and +0.07 V before acquiring data for the positive and negative potential series, respectively.

We base our procedure on the following assumptions: The current flowing through the Pt wire (the CE) is small, so it is reasonable to assume a constant potential at the CE. By definition, the potential of the Pt electrode, i.e. ($E/_{Pt}$), referred to the standard hydrogen electrode potential ($E/_{SHE}$), is given by:

$$E/_{Pt} = E/_{SHE} - E(Pt) \quad (4)$$

where E(Pt) is the Pt potential. Similarly, for the Ag/AgCl reference electrode:

$$E/_{Ag/AgCl} = E/_{SHE} - E(Ag/AgCl) \tag{5}$$

Substituting the expression for ($E/_{SHE}$) from Eq. (4) in Eq. (5) we obtain:

$$E/_{Ag/AgCl} = E/_{Pt} + E(Pt) - E(Ag/AgCl) \tag{6}$$

The potential during the spectra acquisition was applied versus the OCP with no reference electrode, so:

$$E/_{Pt} = E/_{OCP} + OCP \tag{7}$$

Then, combining Eq. (6) and Eq. (7) one obtains:

$$E/_{Ag/AgCl} = E/_{OCP} + OCP + E(Pt) - E(Ag/AgCl) \tag{8}$$

To know the value of E(Pt), we re-measured the OCP, this time using a Ag/AgCl, 3 M KCl reference electrode, without acquiring the THz spectra. We call this new open circuit potential OCP_2. It follows that the potential at the Au WE is:

$$OCP_2 = E(Au) - E(Ag/AgCl) \tag{9}$$

while without RE, it was given by:

$$OCP = E(Au) - E(Pt) \tag{10}$$

From Eq. (10), the expression of E(Pt) can be rewritten using the expression for E(Au) in Eq. (9):

$$E(Pt) = OCP_2 - OCP + E(Ag/AgCl) \tag{11}$$

Substituting Eq. (11) into Eq. (8) we obtain:

$$E/_{Ag/AgCl} = E/_{OCP} + \cancel{OCP} + OCP_2 - \cancel{OCP} + \cancel{E(Ag/AgCl)} - \cancel{E(Ag/AgCl)}$$

i.e.:

$$E/_{Ag/AgCl} = E_{/OCP} + OCP_2 \tag{12}$$

In order to reference the bias potentials in the main text to the Ag/AgCl electrode, one must add the difference between the open circuit reference potential measured in our experiments and the open circuit potential obtained for the Ag/AgCl reference electrode (OCP_2).

Figure S9 shows the result of the OCP_2 measurement in the electrochemical cell filled with 10 mM NaCl solution and composed of the same electrodes used in the experiment plus the Ag/AgCl, 3 M KCl as a RE, constantly purged with Ar gas. Three consecutive measurements have been conducted and, in each case, a stable value was obtained after 3 minutes. Hence, the values recorded at 180 s were used to determine an OCP_2 of ~60 mV as the average of three individual values. The OCP reference potential previously measured (+30 mV and +70 mV for the positive and negative potential series, respectively) can now be referenced to the Ag/AgCl, 3.0 M KCl RE.

We stress here that the conversion of the potential does not change the interpretation of the data.

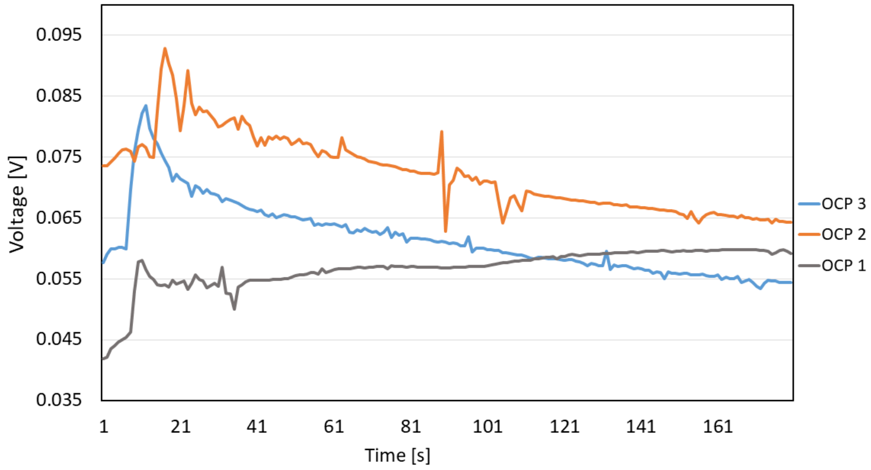

Figure S9: *Measurement of the OCP using a Ag/AgCl reference electrode to determine* OCP_2.

S9. Experimental THz absorption spectra of NaCl solutions

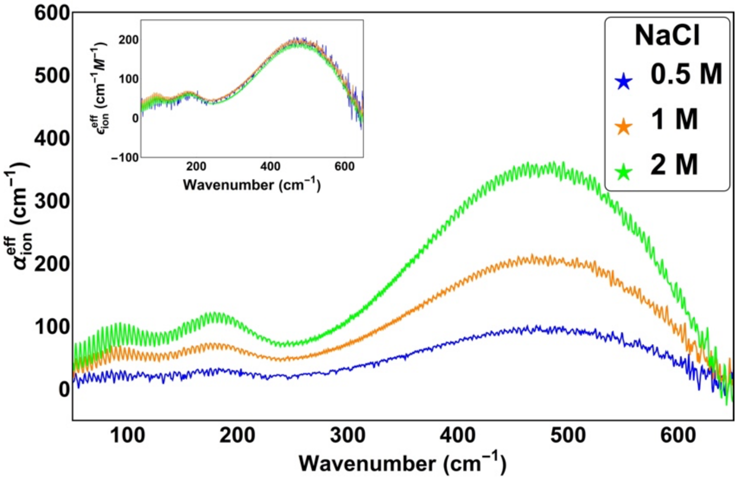

Figure S10: *Experimental THz absorption spectra of NaCl solutions with 0.5, 1, and 2 M concentration. Inset: extinction coefficient (absorption scaled by the salt concentration). We subtracted the pure water spectrum from all three ion absorption spectra and applied a density correction as described in Refs. 38 and 39 of the manuscript.*

In Figure S10 we show the ion specific experimental THz spectrum of the solvated NaCl solutions. It displays cation and anion rattling peaks below 250 cm^{-1} as well as a broad band at higher frequencies. As discussed in more detail in Ref. 39, the latter arises from the dipole-dipole self-correlation of water molecules in the 1st hydration layer and the cross correlations between the dipoles of water molecules in the 1st and 2nd hydration layer. The intensities scale proportionally to the salt concentration, as can be seen in the inset, where we plot the molar extinction coefficient, i.e. the intensity divided by the molar concentration.

S10. Interface definition from MD simulations

For all investigated interfaces, we apply the MD simulation protocol and descriptors we have developed and validated in Ref. 58 in order to define the three relevant interfacial layers: binding interfacial layer (BIL), diffuse layer (DL), and bulk. The EDL region is hence identified as the sum of BIL and DL. The BIL includes the first water layer(s) in contact with the other medium (here the Au surface), which differ from bulk water in terms of water coordination, density and orientation. Within the conditions of the present simulations, the BIL can be identified with the Stern layer in the GCS theory (see Refs. 58,59 for more discussions on the BIL/Stern relationship). The DL is a bulk-like water slab whose thickness can be roughly estimated by the Debye length and corresponds to the reoriented diffuse layer in the GCS theory (at the investigated ionic concentration, see Refs. 58,59). DL-water has similar density and coordination as bulk water, but differs by the net orientation induced by the static field generated by the charged surface. As shown in Ref. 58, three structural descriptors are sufficient to reveal BIL/DL/bulk water layers at all interfaces: water density profiles with respect to the distance from the instantaneous water surface, water coordination (i.e. number of water-water HBs/molecule), and HB orientation heatmaps simultaneously evaluating strength and orientation of water–water HBs. These descriptors are displayed in Figure S11.

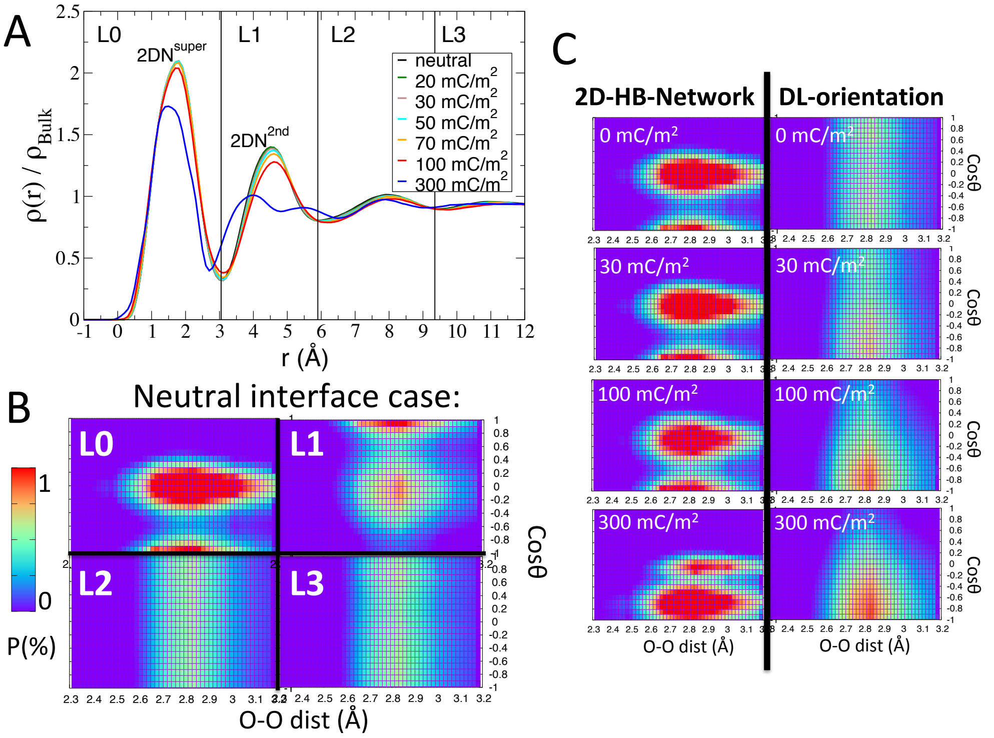

Figure S11: *The interface characterization into BIL and DL layers (which together form the electric double layer) is illustrated for positively charged interfaces as an example.* ***(A)*** *water density profiles* (normalized by the bulk water density) *as a function of the distance from the instantaneous water surface (defined as positive in the liquid phase and negative in the other phase). The vertical dashed lines indicate the L0-L3 layers that can be identified from the maxima in the plot.* ***(B)*** HB orientation heatmaps *of the H-bond patterns formed between the water molecules in each layer. The x-axis reports the O-O distances (Å) between 2 hydrogen-bonded water molecules and the y-axis reports the angles θ between the O-O vector (from donor to acceptor) and the normal to the surface (oriented from liquid to solid). The colors represent the probability (P) to find one O-H group forming one HB with a given distance and orientation. The probability increases from blue to red.* ***(C)*** HB orientation heatmaps *calculated for the L0 layer, where interfacial water forms the 2D-HB network, and for the diffuse layer (DL = L2+L3) as a function of the positive charging of the gold surface. The progressive weakening of the 2D-HB network (disappearance of the red spot at cos(θ)=0) and net orientation of the DL (appearance of the red spot at cos(θ)<0) are well illustrated in the plot. See also Refs. 58,59, where these descriptors and associated plots have been detailed.*

The procedure is illustrated in Figure S11. The water density profiles Figure S11-A are reported for all interfaces with positive σ values (note that identical plots are obtained also for the negatively charged interfaces). All density plots show peaks with decreasing intensity at increasing distance from the surface. Each peak can be associated with a water layer (L0-L3). The density peak in the L0 layer has constant shape and height for all interfaces, with the exception of the highest σ value of 300 mC/m^2 with a lower maximum. Water in the L0 layer is systematically twice as dense as bulk water. It is also found to display an average coordination of 3.0 HBs/molecule (same average value for all 13 surface charges), less than the average value of 3.4 HBs/molecule typical of bulk water and equal to the value obtained for water forming the 2D-HB network at the air-water interface (see Refs. 45,47). Moreover, the HB orientation heatmap for the L0 layer in Figure S11-B shows a red spot at cos(θ) = 0, indicating a preferred orientation parallel to the surface for water-water HBs (2/3 of the total 3.0 HBs/molecule in the layer) formed in this layer. A second red spot is found at cos(θ) = -1, accounting for the remaining 1/3 the total 3.0 HBs/molecule that connects water in L0 with water molecules in the subsequent L1 layer. For all these characteristics, L0 is assigned to the BIL region (see Refs. 47,58,59). Similarly, also the L1 layer has much higher density than bulk, a lower water coordination of 3.1 HBs/molecule and a non-homogeneous distribution of cos(θ) values in the heatmap, different from the homogeneous distribution expected for the bulk water layers (see for example the HB orientation heatmap for L2 and L3 layers shown in Figure S11-B). Thus, L1 is also assigned to the BIL. In particular, the heatmap shows the same red spot at cos(θ) = 0 as observed for L0, indicating that the in-plane water ordering extends to this second layer. The maximum at cosine = 1 identifies the HBs connecting L1 to L0. The existence of a bilayer in the BIL resulting from our analysis is fully consistent with the picture emerging from Ref. 33, where water at the interface with Au was shown to arrange in a topmost adlayer, in direct contact with the Au atoms and characterized by in-plane oriented water-water HBs (our L0), followed by an air-like layer (our L1), where the bulk-like water HB network is not recovered yet. The L2 and L3 layers have a similar density as bulk water (amplitude close to 1 in the density profiles in Figure S11-A) and a water coordination of 3.4 HBs/molecules, as for bulk water. They can hence not be assigned to the BIL anymore (see Ref. 54). Whether they are part of the DL or bulk depends on the orientation distribution in the HB orientation heatmap.

For the neutral interface, the heatmaps (Figure S11-B) show a homogeneous distribution in O-O

angles for the L2 and L3 layers, which are consequently assigned to bulk water, meaning that bulk water follows right after the BIL (which is L0+L1). On the contrary, for all charged interfaces, a DL is observed between the BIL and bulk regions, due to a static electric field that reorients the water molecules generated by the charged surface. The increasing water orientation in the DL as a function of the progressive positive charging of the Au surface can be better appreciated in Figure S10-C. Upon increasing the positive surface charge we observe that the red spot at negative cos(θ) values becomes more prominent, indicating a preferred DL-water orientation with the O-atoms pointing toward the positively charged Au surface and the H-atoms pointing away from it.

S11. 2D-HB network characterization from MD simulations

Based on our MD simulations we observe the 2D-HB network in layer L0 for all investigated surface charges, while a weaker, still relevant in-plane ordering within the HB network also persists in the second L1 layer. The 2D-HB network connects all interfacial water molecules via HBs oriented parallel to the Au surface plane. The strength of the 2D-HB network is measured through the average number of parallel HBs per water molecule forming the 2D-HB network (2DN) (HBs^{2DN} /molecule). Focusing initially on the neutral interface, a connectivity of 2.3 HBs^{2DN}/molecule is observed. This value is much higher than the value of 1.7 previously reported for the 2D-HB network at the air/water interface [45,47]. To understand this difference, the possible configurations of interfacial water molecules need to be evaluated.

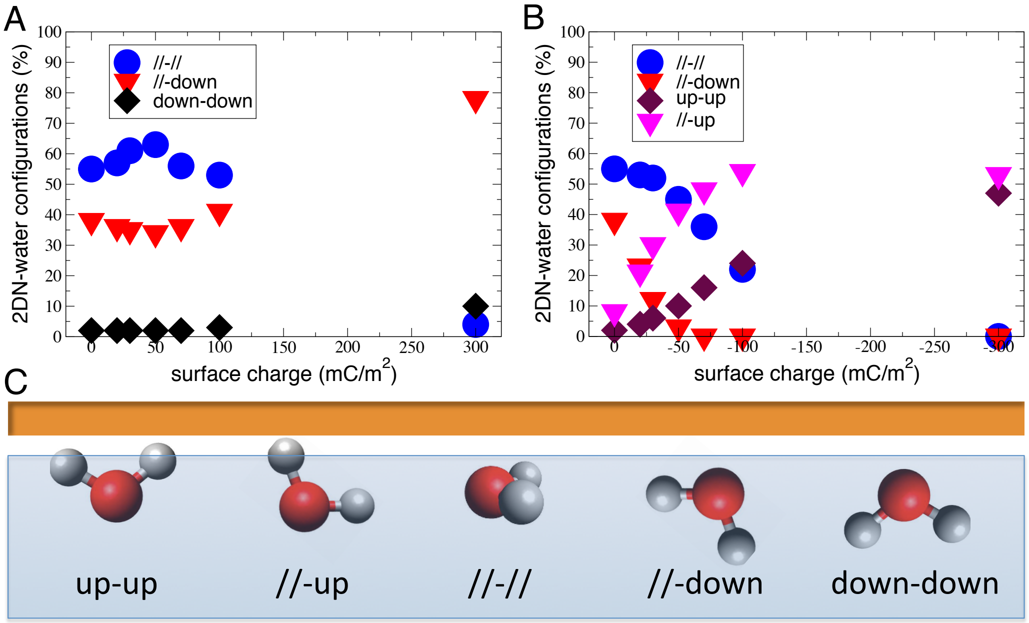

Figure S12: *Probability distribution in terms of the orientation of the two OH-groups of water molecules in 2D-HB network as a function of the positive* ***(A)*** *and negative* ***(B)*** *surface charge. The typical configurations of water are illustrated in panel* ***(C)*** *and are defined from the orientation of the two OH groups of each molecule, where // refers to a water OH group oriented parallel to the gold surface,* ***down*** *to an OH pointing toward the subsequent water layer, and* ***up*** *to an OH pointing to the Au surface. For both the positive and negative charge series, only the water configurations with at least 10% probability observed at one or more σ values are considered in the plots.*

The water molecules forming the 2D-HB network (air/water and Au/water alike) can be classified depending on the orientation of their two OH-groups (Figure S11). For each OH group, three orientations are possible: 1) // for a water OH group oriented parallel to the gold surface, 2) **down** for an OH pointing toward the subsequent water layer, and 3) **up** for an OH pointing to the Au surface. A water having both OH groups with // orientation is therefore labelled //-//, a water with one OH // and the other **down** is labelled **//-down**, and so on. See the illustrations in Figure S11.
At the neutral Au/water interface, the dominant configuration is //-//, in perfect agreement with a recent study [19], while the //-**down** population was found to be the most probable at the air/water interface [45,47]. The //-// water molecules contribute to the 2D-HB network with either 2 or 3 HBs^{2DN}/molecule, one more than the average contribution of //-**down** waters (either 1 or 2

HBs^{2DN}/molecule). This is why the number of HBs^{2DN}/molecule is higher than 2 at the Au/water interface and lower than 2 at the air/water interface. The //-// configuration is favoured at the Au/water interface due to interactions between the water O-atom and Au atoms, which template the in-plane ordering within the interfacial water layer, as also discussed in Ref. 33.

S12. Ions hydration shell characterization from MD simulations

The average ion-water coordination number is calculated from the MD simulations by counting the average number of water molecules (identified by their oxygen atoms) which are found at a distance from the ions lower than the first minimum of the ion-water (i.e. Cl-O/Na-O) radial distribution function in bulk water. This minimum is 3.8 Å for Cl-O and 3.2 Å for Na-O. The average coordination number of both ions in the Outer Helmholtz Plane (OHP) is found to be equal to the one observed in the DL/bulk region. It is equal to 6.11 ± 0.89 for the Cl^- and 5.60 ± 0.64 for the Na^+. On the contrary, inner sphere adsorbed ions populating the Inner Helmholtz Plane (IHP) are less coordinated than ions in the bulk and loose on average one hydration water. The Cl-O and Na-O coordination numbers in the IHP are 4.78 ± 0.93 and 4.83 ± 0.33 respectively.

However, these coordination numbers are averages, resulting from the statistical distribution of all possible instantaneous coordination numbers of the ions over time. The associated error bars are a measure of the broadness of such a distribution. A more detailed analysis of the ions hydration shell can be achieved by evaluating the probability distribution of both Na-O and Cl-O coordination numbers.

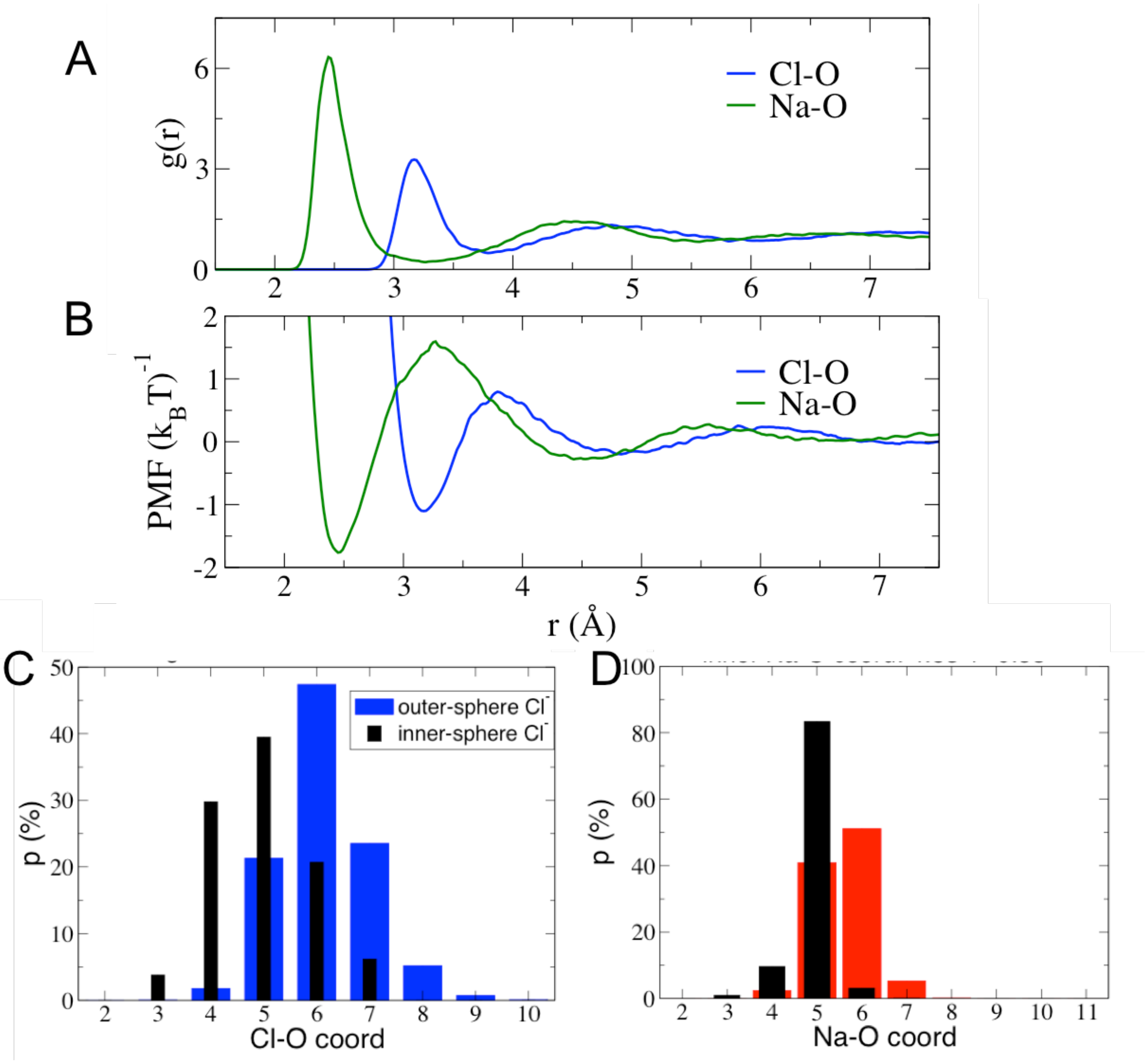

Figure S13: *(**A**): Radial pair distribution function (g(r)) between all Na^+ and Cl^- ions and all water O-atoms. (**B**) Corresponding potential of mean force (PMF, in units of k_BT) to extract a water molecule from the hydration layer of the two ions. **(C-D)**: Probability distribution of the coordination numbers for Cl^- **(C)** and Na^+ **(D)** in the OHP (black) and in the IHP (blue/red). The ion coordination numbers in the OHP are equal to the reference coordination numbers in the bulk.*

This is done in Figure S13 for both cations and anions located in the IHP and in the OHP, by evaluating the probability that each possible coordination number is indeed observed during the simulation. In the OHP, all coordination numbers between 4 and 9 are observed for Cl^-, while all numbers between 4 and 7 are observed for Na^+. For both ions, the most probable coordination

number in the OHP/bulk is found to be 6. It becomes 5 in the IHP, hence the coordination number distribution for both ions is decreased by one water molecule.

Na^+ is expected to be more anchored to its hydration waters due to its higher charge density compared to Cl^- (48). Based on this consideration, the intrinsic differences in desolvation free energies, i.e., the free energy cost to remove a single water molecule from the solvation shell of Na^+ vs Cl^-, would also contribute to asymmetries in the double layer structure (and the composition of the IHP in particular) for positive and negative electrodes. In order to investigate such a possibility, we calculated the potential of mean force (PMF) to remove one hydration water from the hydration layer of Cl^- and Na^+ from the MD simulations. The PMF (Figure S13-B) is directly deduced from the ion-water radial pair distribution function g(r) (Figure S13-A) as –ln(g(r)). For both ions, the free energy barrier to remove one water molecule from the hydration shell can be estimated as the free energy difference between the 1st minimum (at ~2.5 Å for Na^+ and ~3.1 Å for Cl^-) and the subsequent maximum (at ~3.2 Å for Na^+ and ~3.8 Å for Cl^-). The calculated barrier is ~3.4 k_BT for Na^+, larger than the value of ~1.8 k_BT obtained for Cl^-. Based on the difference in the free energy cost to remove a water molecule from the hydration shells of the two ions one would expect that the Cl^- hydration layer is more easily stripped off than the Na^+ hydration layer. This is the opposite of what we observe at the Au/water interface, confirming that other free energy terms are responsible for the asymmetry in the ion adsorption and solvation at the interface.

S13. Theoretical Free Energy profiles to understand the ions adsorption at the Au surface

The free energy profiles to bring cations or anions from the bulk to the interface have been estimated by:

$$\Delta G(r) = -k_B T \ln \frac{P(r)}{P_{bulk}} \tag{13}$$

where $\Delta G(r)$ is the free energy required to bring the ion from the bulk to a given distance from the surface, *P(r)* is the probability for the ion to be located at the distance *r* from Au, and is normalized here by the probability to be located in the bulk P_{bulk}. *P(r)* and P_{bulk} are readily calculated from the MD simulations, just by counting the average number of ions found at each distance *r* from the Au surface along the MD trajectories. This is legitimate in classical MD simulations once proper conformational sampling is achieved.

The resulting profiles are shown in Figure S14 for Na^+ for the MD trajectory at the negative surface charge of -100 mC/m^2 and for Cl^- at the positively charged surface of σ= +100 mC/m^2. This choice allows to clearly separate both minima related to the IHP (around 3 Å) and to the OHP (around 6-7 Å). The minima are less pronounced at lower surface charge values. Please note that any other choice for σ in the ±20-100 mC/m^2 range, where the ions adsorption process is still dominated by their interaction with the 2D-HB network (see main text and previous sections in the SI), does not change the final conclusions obtained from this analysis.

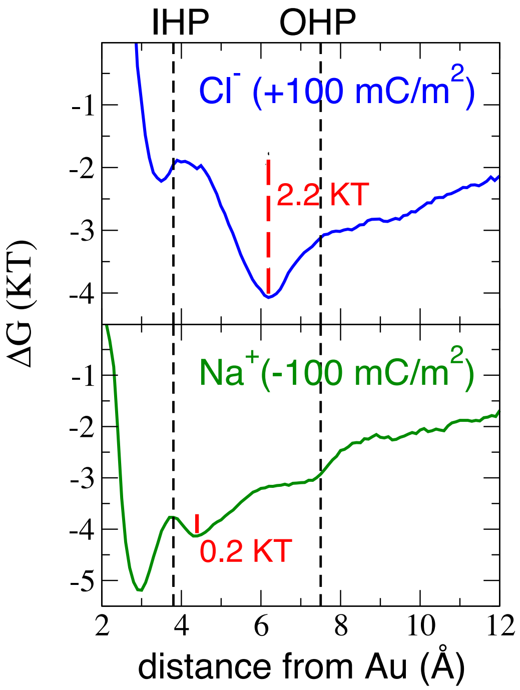

Figure S14: *Theoretical free energy profiles of* Cl^- *(up, blue) and* Na^+ *(down, green) as a function of the vertical distance from the Au surface (z-axis coordinate), calculated and illustrated here at ± 100 mC/m*^{2} *surface charge conditions. The limits of the IHP and OHP are marked by vertical dashed lines. The calculated free energy barriers to move the ions from the OHP to the IHP are hence equal to 2.2* k_BT *and 0.2* k_BT *for* Cl^- *and* Na^+ *respectively.*

The plots show that, to move Cl^- from the OHP to the IHP, one has to pay a rather high free energy cost of 2.2 k_BT (red vertical dashed line in the figure), while the corresponding cost is reduced to only 0.2 k_BT in the Na^+ case. As explained in the main text, this is due to two simultaneous decisive factors:

1) **water-ion interactions.** The ions lose one hydration water when going from the OHP to the IHP.
2) **water-water interactions.** The Cl^- anions located in the OHP strengthen the 2D-HB network by increasing the number of water-water HBs formed parallel to the surface up to 0.3 HBs/molecule (see section S9), whereas this is not the case for the Na^+ cations. This growth favours the Cl^- location outside the 2D-HB network and explains in part the higher free energy barrier for the Cl^- anion migration from the OHP to the IHP due to the concomitant disruption of the 2D-HB network.

We want to stress here that the values obtained for the free energy barriers should not be considered a quantitative estimate of the energetics associated with ion adsorption at the Au surface, due to the limitations of the present classical MD simulations (see sections S14-S18 for more detailed discussions). However, the order of magnitude difference between the free-energy barriers to bring Cl^- and Na^+ in the IHP do provide a clear qualitative rationalization for the decisive role of both ion-water and water-water interactions for the EDL formation.

S14. Computational details for electronic-structure calculations on cluster models

All density functional calculations for the cluster models were carried out with the Q-Chem electronic structure package [53]. We applied the RPBE functional [54] for consistency with the periodic slab calculations and the ωB97X-V functional [55] to analyze how an accurate hybrid functional compares with RPBE. For all structure optimizations we employed the def2-SVPD basis set [60] with the corresponding def-ECP pseudo-potential for the gold atoms. For the energy decomposition analysis, we additionally used a larger def2-TZVP basis. We used the second-generation energy decomposition analysis [61] and adiabatic energy decomposition analysis [62]. Initial structures for the microsolvation analysis were generated with CREST [63] and the GFN2-xTB tight-binding method [64] as the electronic structure backend. We then reoptimized these structures with ωB97X-V/def2-SVPD.

S15. Computational Details for Electronic Structure Periodic Slab Calculations on Periodic Slab Models

All density functional theory calculations for the periodic slab models were carried out on the Au (100) surface using the Vienna Ab Initio Simulation Package (VASP) [56,57] with the revised Perdew−Burke−Ernzerhof (RPBE) functional developed by Hammer et al. [61] and the projector augmented-wave (PAW) method [65,66]. The surface unit cell (periodically extending in the *x*- and *y*-directions) was designed as four layers of eight Au atoms, with a lattice constant of 4.0782 Å [67] and 20 Å spacing (in the *z*-direction) between two images. A *k*-point sampling of the Brillouin zone was achieved using the 4 × 4 × 1 Monkhorst−Pack mesh, and 500 eV and 0.1 eV were used as the plane-wave cut off energy and the Fermi-smearing width, respectively. Geometries of adsorbates (Na^+, Cl^-, and water) were optimized until the electronic energy and the atomic force were smaller than 10^{-6} eV and 0.05 eV/Å, respectively.
The implicit aqueous solvent and the electrolyte were implemented with a dielectric constant $\varepsilon_r =$ 78.4 and a Debye–Hückel length $\lambda_b = 9.61$ Å (equivalent to a 0.1 M concentration of electrolyte) using the constant electrode potential (CEP) model in the VASPsol patch of VASP [68,69]. The linearized Poisson−Boltzmann equation

$$\varepsilon_r(\nabla^2 - \lambda_b^{-2})\phi(\boldsymbol{r}) = 4\pi[\rho_{\mathrm{DFT}}(\boldsymbol{r}) + \rho_{\mathrm{ext}}(\boldsymbol{r})] \tag{14}$$

was applied to address the effect of the surface charge. Here the potential field (ϕ(r)) was updated self-consistently based on the charge density of the DFT system ($\rho_{\mathrm{DFT}}(\boldsymbol{r})$) and the electrical double layer ($\rho_{\mathrm{ext}}(\boldsymbol{r})$). $\phi(\mathbf{r})$ was later used in the Kohn−Sham equation as the effective potential. The total number of electrons in the unit cell, which can be fractional, was also updated self-consistently so that the Fermi level (E_F, or the chemical potential) of the system satisfied

$$-eU_{\mathrm{SHE}} = E_F + \phi_{\mathrm{SHE}} \tag{15}$$

where the applied electrode potential (U_{SHE}) with respect to the standard hydrogen electrode (SHE) reached a fixed constant. The work function of ϕ_{SHE} = 4.43 eV [70] was used as theoretically predicted for the RPBE functional. In the results presented here, U_{SHE} was converted U_{Au}, the electrode potential with respect to the computational Au electrode (defined when the net charge on the gold slab is zero) through a linear relation:

$$U_{\mathrm{Au}} = U_{\mathrm{SHE}} - 0.12417\ \mathrm{V} \tag{16}$$

S16. Energy Decomposition Analysis of Ion Binding on Charged Metal Clusters

In order to study the nature of the ion bonding interactions with gold in more detail based on electronic structure calculations, we constructed charged Au cluster models that allow us to use more accurate density functionals than the generalized gradient approximation (GGA) functionals that can be employed in the periodic slab calculations. We started from the Au_9^+ cluster from Ref. 70 and reoptimized the structure with both the RPBE and the ωB97X-V functional, which gave no qualitative structural differences. We then reoptimized the structure for all molecular charges ± 2, ± 1, and 0. For both positive charged clusters and the neutral cluster, we arrived at a similar structure than the one from Ref. 71 (Figure S15-A), whereas the optimization for the negatively charged clusters resulted in the structure shown in Figure S15-B.

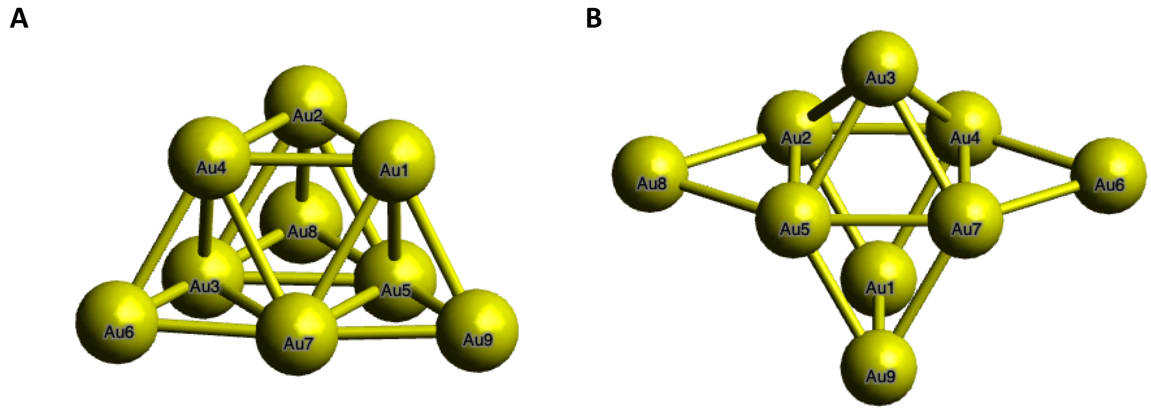

Figure S15: *Au_9 cluster models used in this study. Panel A) is the global minimum for Au_9^+ (see Ref. 73) and is a local minimum in the neutral and dicationic case. Structure optimizations for the anionic Au_9^- and dianionic Au_9^{2-} resulted in the structure shown in Panel B) for both the RPBE and the ωB97X-V functional. Analyses for the Au-Na interaction are therefore based on the cluster model in B), whereas the Au-Cl interaction is modelled with the cluster model in A).*

We then analysed the preferred binding site of Cl^- on the positively charged cluster and Na^+ on the negatively charged cluster. We started from five different initial structures that were generated by placing the ion on different atop-, bridged- or hollow-type sites. For both the RPBE and the ωB97X-V functional, the preferred binding site of Cl^- was an atop site on the most reactive (least

coordinated) Au atom (Figure S16-A), whereas Na^+ prefers a hollow-type site, coordinating four gold atoms almost equally (Figure S16,-B). Both binding modes are very similar to the preferred binding sites identified in the periodic slab calculations (Figure S21). Since the structure of the neutral cluster resembles the positively charged ones, we add the bonding of Cl^- to the neutral cluster in our analysis but refrain from doing this for Na^+ because the large structural changes between the neutral and the negatively charged clusters make a comparison impossible.

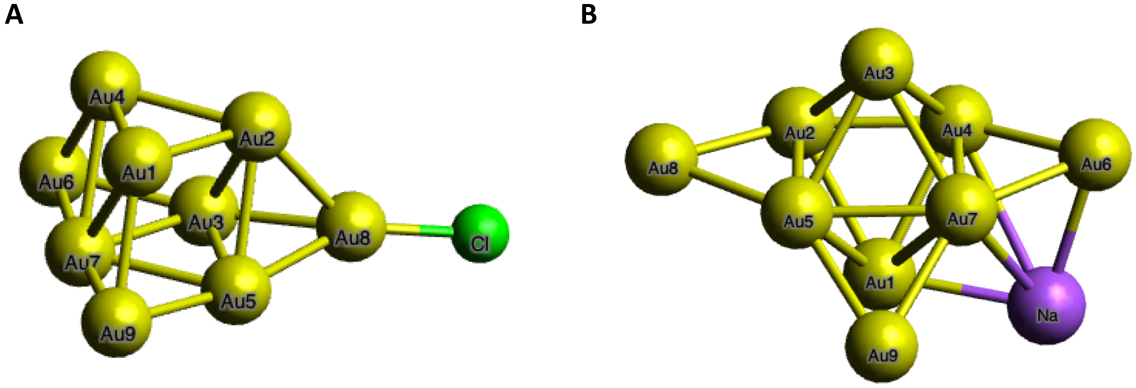

Figure S16: *Panel A) shows the preferred binding site of* Cl^- *for the neutral, cationic and dicationic case. It binds to the most reactive and undercoordinated site. The preferred binding site of* Na^+ *to the anionic and dianionic cluster model is shown in Panel B).*

The energy decomposition analysis (EDA) allows us to split the binding energy of the neutral and positively charged complexes with the chloride ion into three different physically motivated terms. The "frozen" term describes the electrostatic interaction and Pauli repulsion (and dispersion in case of ωB97X-V) between the fragments. The "polarization" term describes the energy lowering due to relaxation of the fragment orbitals in the presence of orbitals on the other fragment. Finally, the "charge-transfer" term describes electron exchange between the fragments.

The frozen term for the chloride atom at the neutral cluster is positive due to Pauli repulsion and the difference between RPBE and ωB97X-V can be explained by the absence of dispersion in the former functional (Figure S17-A). The presence of the charged ion clearly induces a compensating image charge at or near the binding site of chloride on the metal cluster which is manifested in the sizable polarization term. The charge-transfer term then indicates the donation of about 0.05 of an

electron from the chloride anion to the metal cluster. Upon charging the cluster, the frozen term becomes strongly binding due to the increased electrostatic interaction, whereas the polarization and charge-transfer terms are nearly constant. (Figure S17-B and Figure S17-C).

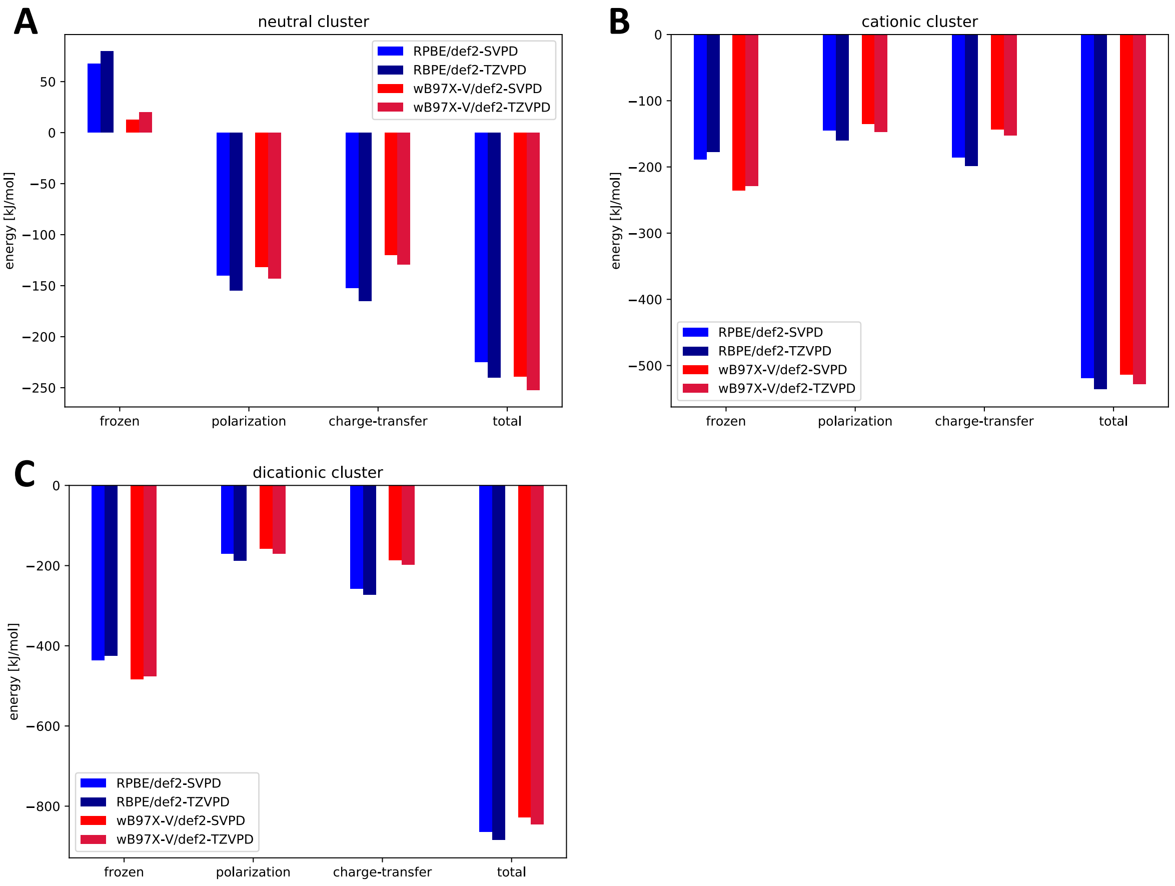

Figure S17: *Energy components resulting from the energy decomposition analysis for the chloride ion bound to a neutral (A), cationic (B), or dicationic (C) Au_9 cluster. The structure of the gold cluster is not relaxed in these calculations ("vertical" EDA). The blue bars correspond to the RPBE density functional, whereas the red bars correspond to the ωB97X-V density functional. Brighter colours denote results obtained with the def2-SVPD basis set and darker colours correspond to the larger def2-TZVPD basis set.*

An instructive illustration of the effect of the relative energy contributions on the binding is the variation of the bond lengths between fragments based on adiabatic EDA, where the structure is optimized at the frozen (FRZ), polarized (POL) and full (FULL) levels of theory. The Au-Cl bond

lengths, corresponding to Au8-Cl in Figure S16-A, are displayed in Figure S18 for the cationic and dicationic cluster. The bond lengths corresponding to the calculations denoted as FULL are the unconstrained optimization results. Those denoted POL correspond to a potential energy surface where charge transfer between the fragments is not permitted and finally FRZ optimizes the structure on a potential energy surface where neither orbital relaxation due to fragment interaction nor charge transfer is allowed. The results clearly show that polarization and charge-transfer have a profound influence on the bond distance for these charged cluster models. Again, the difference between RPBE and ωB97X-V in the first term can be attributed to the lack of dispersion interaction in RPBE.

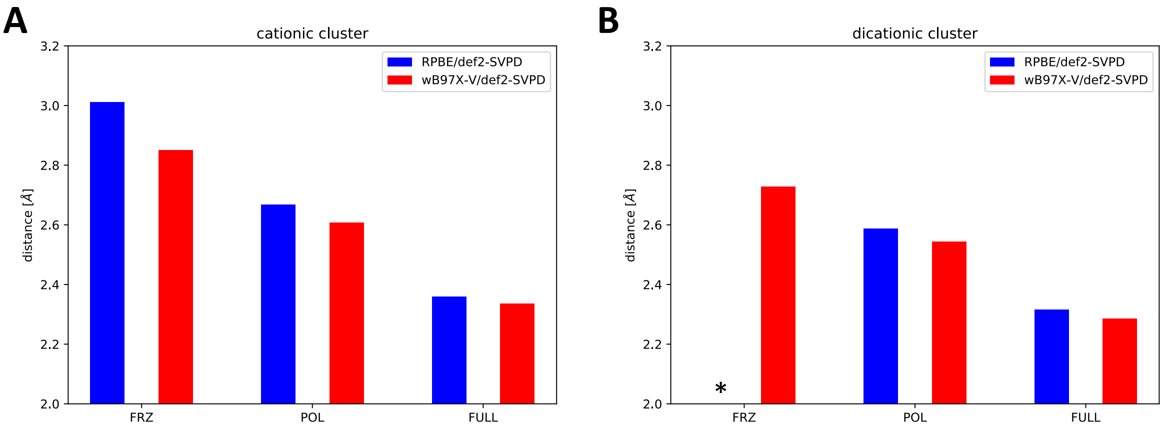

Figure S18: *Au-Cl bond lengths resulting from the adiabatic energy decomposition analysis for the cationic and dicationic gold cluster. The bond length is measured with respect to Au8 in Figure S16-A. Bond lengths denoted as FULL correspond to an unconstrained optimization, those denoted as POL do not include charge-transfer contributions and those denoted as FRZ include neither polarization nor charge-transfer. The missing data point indicated with an asterisk corresponds to a distorted structure that makes a comparison with the other bond lengths impossible.*

The bonding picture obtained from EDA is different for Na^+ with the anionic cluster models. While the electrostatic interaction is slightly stronger for sodium at its preferred hollow site than for the atop bound chloride ion, the polarization term is of similar magnitude. Charge-transfer from the anionic cluster to the sodium ion, however, is completely absent in stark contrast to the chloride bound cluster models.

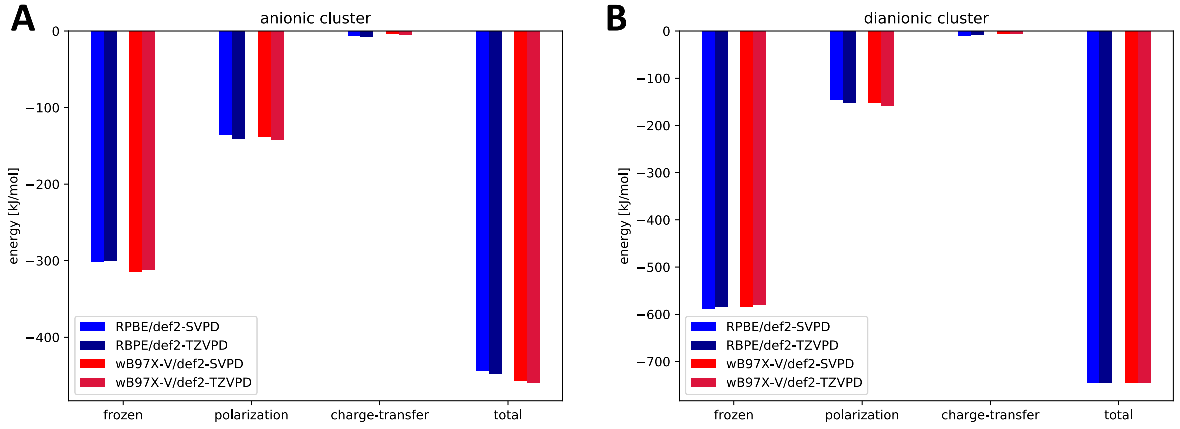

Figure S19: *Energy components resulting from the energy decomposition analysis for the sodium ion bound to an anionic (A or dianionic (B) Au_9 cluster. The structure of the gold cluster is not relaxed in these calculations. The blue bars correspond to the RPBE density functional, whereas the red bars correspond to the ωB97X-V density functional. Brighter colours denote results obtained with the def2-SVPD basis set and darker colours correspond to the larger def2-TZVPD basis set.*

This is clearly reflected in the bond distances calculated from the adiabatic EDA analysis. Note that the bond lengths are now averaged over the four gold atoms closest to Na^+ (Au 1,4,6,7 in Figure S16-B). While polarization shortens the bond length to a similar extent as for Cl^-, charge-transfer does not affect the structure at all.

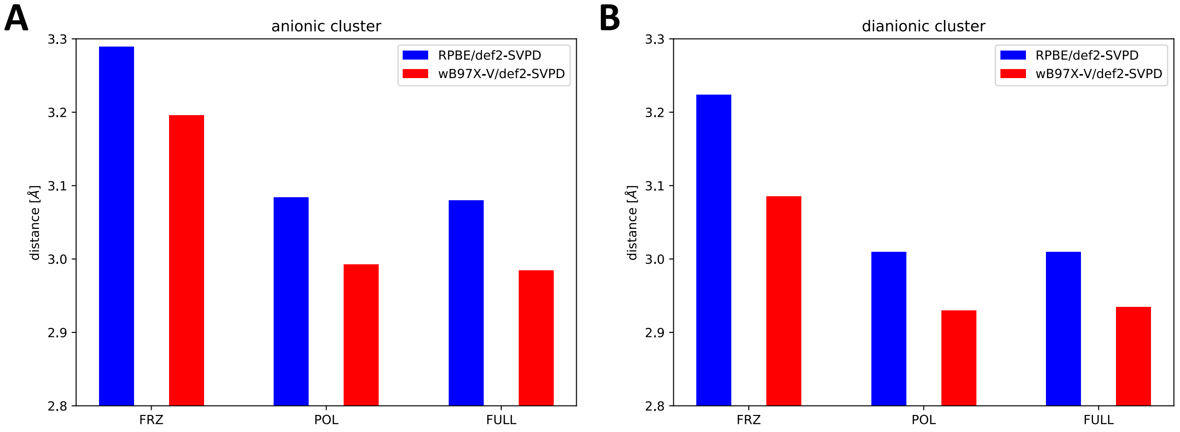

Figure S20: *Au-Na bond lengths resulting from the adiabatic energy decomposition analysis for the anionic and dianionic gold cluster. The bond length shown is the average of the Na-Au distance for Au(1,4,6,7) in Figure S16-B. Bond lengths denoted as FULL correspond to an unconstrained optimization, those denoted as POL do not include charge-transfer contributions and those denoted as FRZ include neither polarization nor charge-transfer.*

S17. Effect of Applied Bias on Ion Binding and Electrode Polarization

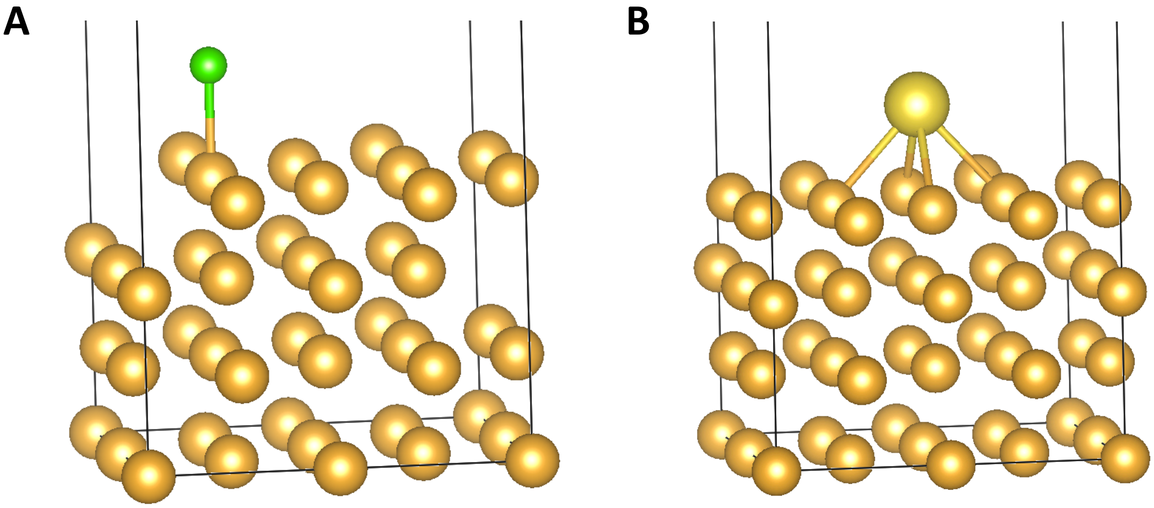

Figure S21: *Optimized structures from the grand-canonical periodic slab calculations with implicit solvation. For all applied bias potentials, Cl^- prefers an atop binding mode (panel A), whereas Na^+ prefers binding to a hollow site (panel B).*

As mentioned above, the fixed chemical potential, periodic slab calculations predict a preferred binding of Cl^- to an atop site and a hollow site binding of Na^+ on the Au(100) surface (Figure S21). Based on these initial structures at the potential of zero charge, we optimize the structures for different bias potentials, keeping the lowest two Au layers fixed while the top two layers are fully relaxed.

In Table S2 we analyse the effect of the bias voltage on the bond length, the total number of electrons in the system in presence and absence of the ions, the net charge, the change in partial charge of the binding Au atoms upon ion binding, and the partial charge of the ions themselves. The partial charge information is calculated from a Bader analysis of the converged grand-canonical calculations. A slight shortening of the ion-gold distance is observed upon charging the slab model as expected due to the increased electrostatic interaction. While the net charge of the metal slab is clearly a function of the applied bias, the change of the number of electrons in the presence of the bound ion is virtually constant in case of Na^+ and varies only very slightly for Cl^-. This is also true for the local charge on the Au atom(s) closest to the ion. We hence conclude that the polarization of the electrode upon ion binding is independent of the bias voltage in this range and can therefore not explain the bias dependent differences observed experimentally. These results accordingly suggest that the neglect of polarization and charge-transfer in the classical MD simulations should not materially impact the simulation results, or the interpretation of the experimental data in light of the simulations.

Table S2: *Summary of the slab calculation results with one ion (Na^+ or Cl^-). The applied bias potential is given with respect to the computational gold electrode as defined in* ***S16****. The table shows the distance d of the ions to the gold surface, the change in the electron number upon ion binding, the net charge of the surface when no ion is present and changes in the local charge of the bonding Au atom(s) as calculated by a Bader analysis.*

a) Since Na^+ favours a hollow site binding mode, d refers to the distance to the plane defined by the four closest Au atoms.

b) The value refers to the four closest Au atoms.

c) Since Cl- prefers an atop site binding mode, d refers to the distance to the Au atom it binds to.

d) The value refers to the closest Au atom.

	Na^+					
bias (vs. U_{Au}) / V	0.0	-0.1	-0.2	-0.3	-0.5	-1.0
d (Au-Na^+) / Å [a)]	2.21	2.21	2.21	2.20	2.17	2.13
$\sum N_{Au}^{(Na^+)} - \sum N_{Au}^{pure}$	0.42	0.42	0.41	0.41	0.41	0.42
net charge (no ion) / e	0.00	-0.10	-0.20	-0.29	-0.48	-0.91
Δ local charge Au / e [b)]	-0.04	-0.04	-0.04	-0.04	-0.04	-0.05
local charge Na^+	0.005	0.005	0.005	0.006	0.006	0.007
	Cl^-					
bias (vs. U_{Au}) / V	0.0	0.1	0.2	0.3	0.5	1.0
d (Au-Cl^-) / Å [c)]	2.40	2.40	2.40	2.39	2.39	2.36
$\sum N_{Au}^{(Cl^-)} - \sum N_{Au}^{pure}$	-0.34	-0.33	-0.32	-0.30	-0.27	-0.18
net charge (no ion) / e	0.00	0.10	0.21	0.32	0.54	1.10
Δ local charge Au / e [d)]	0.15	0.14	0.14	0.13	0.12	0.07
local charge Cl^-	-0.58	-0.57	-0.57	-0.56	-0.54	-0.52

S18. Microsolvation Analysis for Na^+ and Cl^- based on Quantum Chemical Calculations

In order to investigate the preferred explicit water solvation environment for Na^+ and Cl^-, we performed a conformer search for both ions with 6, 8, 10, and 12 water molecules with the GFN2-xTB density functional tight-binding method [64] and the CREST program [63]. We reoptimized the 3 lowest conformers of each calculation with ωB97X-V/def2-SVPD. The global minima for both ions with 6 and 12 water molecules are displayed in Figure S22. As observed previously in studies investigating the ion-pairing of NaCl (Ref. 72), Na^+ prefers a symmetrical, almost tetrahedral environment, whereas Cl^- prefers an asymmetric, surface-like structure with 4-5 water molecules in the first hydration shell. The 2D-HB network we identified from our classical MD simulations and that has been described elsewhere (e.g. Ref. 33), can be described as hydrophobic and separates the bulk water in the adjacent layer. The OHP is therefore ideal to accommodate the chloride anions in their preferred solvation environment while keeping the 2D-HB network uninterrupted and even strengthening it by attracting bulk water molecules that would otherwise be engaged in hydrogen bonds with the first water layer. Only when the electrostatic interaction strength between Cl^- and the Au surface reaches a certain threshold, it becomes favourable for these ions to strip off the hydration shell and bind directly to the surface.

In contrast, the symmetric environment preferred by sodium ions, can be realized already at low bias potentials in the IHP when one or two water molecules that bind to Na^+ are replaced by the negatively charged gold atoms on the surface. There is therefore no preferred solvation pattern that needs to be sacrificed for the binding to the gold surface in case of Na^+ which causes the 2D-HB network to be disrupted already for low negative bias potentials.

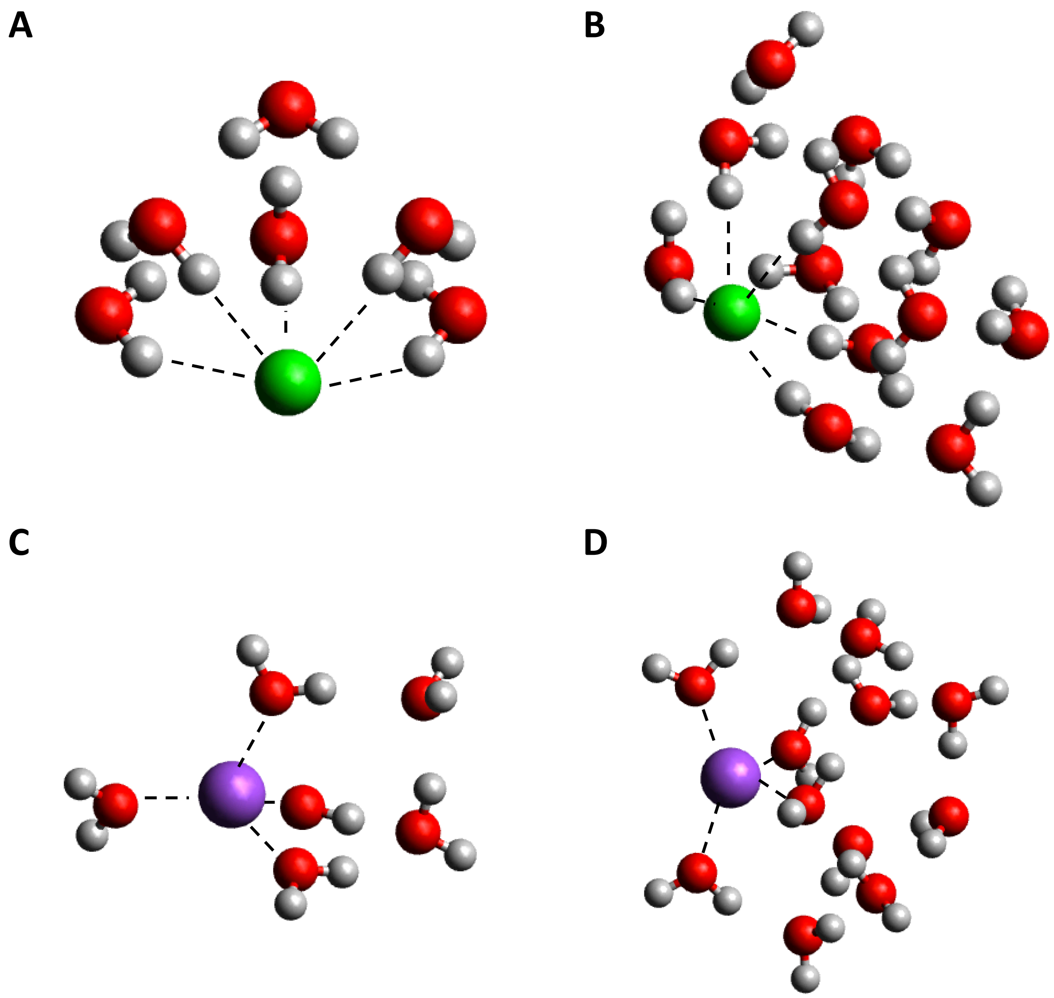

Figure S22: *Optimized global minima for microsolvated ion structures. Panels A) and B) show the microsolvated Cl^- structures with 6 and 12 H_2O molecules respectively, whereas panels C) and D) show the microsolvated Na^+ structures with 6 and 12 H_2O molecules, respectively. Cl^- clearly prefers asymmetric solvation, where one half-plane is uncoordinated, whereas Na^+ prefers symmetric solvation with an almost tetrahedral first coordination shell.*

References for the SI

[58] S. Pezzotti, D. R. Galimberti, Y. R. Shen, M.-P. Gaigeot, Structural definition of the BIL and DL: a new universal methodology to rationalize non-linear χ(2)(ω) SFG signals at charged interfaces, including χ(3)(ω) contributions. Phys. Chem. Chem. Phys. 20, 5190–99 (2018).

[59] S. Pezzotti, D. R. Galimberti, M.-P. Gaigeot, Deconvolution of BIL-SFG and DL-SFG Spectroscopic Signals Reveal Order/Disorder of Water at the Elusive Aqueous Silica Interface. Phys. Chem. Chem. Phys. 21, 22188-22202 (2019).

[60] F. Weigend, R. Ahlrichs, Balanced basis sets of split valence, triple zeta valence and quadruple zeta valence quality for H to Rn: Design and assessment of accuracy, Phys. Chem. Chem. Phys., 7, 3297-3305 (2005).

[61] P. R. Horn, Y. Mao, M. Head-Gordon, Probing non-covalent interactions with a second generation energy decomposition analysis using absolutely localized molecular orbitals, Phys. Chem. Chem. Phys. 18, 23067-79 (2016).

[62] Y. Mao, P.R. Horn, M. Head-Gordon, Energy decomposition analysis in an adiabatic picture, Phys. Chem. Chem. Phys. 19, 5944-58 (2017).

[63] S. Grimme, Exploration of Chemical Compound, Conformer, and Reaction Space with Meta-Dynamics Simulations Based on Tight-Binding Quantum Chemical Calculations, J. Chem. Theory Comput. 15, 2847-62 (2019).

[64] C. Bannwarth, S. Ehlert, S. Grimme, GFN2-xTB — An Accurate and Broadly Parametrized Self-Consistent Tight-Binding Quantum Chemical Method with Multipole Electrostatics and Density-Dependent Dispersion Contributions, J. Chem. Theory Comput. 15, 1652-71 (2019).

[65] P. E. Blöchl, Projector augmented-wave method, Phys. Rev. B 50, 17953-979 (1994).

[66] G. Kresse, D. Joubert, From ultrasoft pseudopotentials to the projector augmented-wave method, Phys. Rev. B 59, 1758-75 (1999).

[67] A. Maeland, T. B. Flanagan, Lattice Spacings of Gold–Palladium Alloys, Canad. J. Phys. 42, 2364-66 (1964).

[68] K. Letchworth-Weaver, T. A. Arias, Joint density functional theory of the electrode electrolyte interface: Application to fixed electrode potentials, interfacial capacitances, and potentials of zero charge, Phys. Rev. B 86 , 075140 (2012).

[69] J. D. Goodpaster, A. T. Bell, M. Head-Gordon, Identification of possible pathways for C−C bond formation during electrochemical reduction of CO2: New theoretical insights from an improved electrochemical model, J. Phys. Chem. Lett. 7, 1471-77 (2016).

[70] R. Jinnouchi, A. B. Anderson, Aqueous and surface redox potentials from self-consistently determined Gibbs energies, J. Phys. Chem. C 112, 8747-50 (2008).

[71] A. A. A. Atia, A. M. V. Branzanic, A. Munoz-Castro, A. Lupan, R. Bruce King, Cationic gold clusters with eight valence electrons, Phys. Chem. Chem. Phys. 32, 17779-85 (2019).

[72] G.-L. Hou, C.-W. Liu, R.-Z. Li, H.-G. Xu, Y. Q. Gao, W.-J. Zheng, Emergence of Solvent-

Separated Na+-Cl- Ion Pair in Salt Water: Photoelectron Spectroscopy and Theoretical Calculations, J. Phys. Chem. Lett. 8, 13 (2017).
